# Phylogenetic conservation of Trop-2 across species—rodent and primate genomics model anti-Trop-2 therapy for pre-clinical benchmarks

**DOI:** 10.3389/fgene.2023.1297367

**Published:** 2024-01-05

**Authors:** Emanuela Guerra, Marco Trerotola, Valeria Relli, Rossano Lattanzio, Khouloud Boujnah, Nicole Travali, Antonino Moschella, Paolo Todaro, Laura Pierdomenico, Roberta Di Pietro, Nicola Tinari, Saverio Alberti

**Affiliations:** ^1^ Laboratory of Cancer Pathology, Center for Advanced Studies and Technology (CAST), “G. d’Annunzio” University of Chieti-Pescara, Chieti, Italy; ^2^ Department of Medical, Oral and Biotechnological Sciences, “G. d’Annunzio” University of Chieti-Pescara, Chieti, Italy; ^3^ Department of Innovative Technologies in Medicine and Dentistry, “G. d’Annunzio” University of Chieti-Pescara, Chieti, Italy; ^4^ Unit of Medical Genetics, Department of Biomedical Sciences—BIOMORF, University of Messina, Messina, Italy; ^5^ Department of Human Pathology “Gaetano Barresi”, Section of Cytopathology, University of Messina, Azienda Ospedaliera Universitaria “Gaetano Martino”, Messina, Italy; ^6^ Department of Medicine and Aging Sciences, Center for Advanced Studies and Technologies (CAST), “G. d’Annunzio” University of Chieti-Pescara, Chieti, Italy; ^7^ Department of Medicine and Aging Sciences, Section of Biomorphology, “G. d’Annunzio” University of Chieti-Pescara, Chieti, Italy; ^8^ Sbarro Institute for Cancer Research and Molecular Medicine, Center for Biotechnology, Department of Biology, College of Science and Technology, Temple University, Philadelphia, PA, United States

**Keywords:** Trop-2, phylogenetic conservation, pharmacokinetics, toxicity, primate genomics

## Abstract

A phylogenetic conservation analysis of Trop-2 across vertebrate species showed a high degree of sequence conservation, permitting to explore multiple models as pre-clinical benchmarks. Sequence divergence and incomplete conservation of expression patterns were observed in mouse and rat. Primate Trop-2 sequences were found to be 95%–100% identical to the human sequence. Comparative three-dimension primate Trop-2 structures were obtained with AlphaFold and homology modeling. This revealed high structure conservation of Trop-2 (0.66 ProMod3 GMQE, 0.80–0.86 ± 0.05 QMEANDisCo scores), with conservative amino acid changes at variant sites. Primate *TACSTD2/TROP2* cDNAs were cloned and transfectants for individual ORF were shown to be efficiently recognized by humanized anti-Trop-2 monoclonal antibodies (Hu2G10, Hu2EF). Immunohistochemistry analysis of *Macaca mulatta* (rhesus monkey) tissues showed Trop-2 expression patterns that closely followed those in human tissues. This led us to test Trop-2 targeting *in vivo* in *Macaca fascicularis* (cynomolgus monkey)*.* Intravenously injected Hu2G10 and Hu2EF were well tolerated from 5 to 10 mg/kg. Neither neurological, respiratory, digestive, urinary symptoms, nor biochemical or hematological toxicities were detected during 28-day observation. Blood serum pharmacokinetic (PK) studies were conducted utilizing anti-idiotypic antibodies in capture-ELISA assays. Hu2G10 (t_1/2_ = 6.5 days) and Hu2EF (t_1/2_ = 5.5 days) were stable in plasma, and were detectable in the circulation up to 3 weeks after the infusion. These findings validate primates as reliable models for Hu2G10 and Hu2EF toxicity and PK, and support the use of these antibodies as next-generation anti-Trop-2 immunotherapy tools.

## 1 Introduction

Trop-2 is a Ca^2+^-signal transducer (Ripani et al., 1998) and a tumor and stem cell growth inducer (Stoyanova et al., 2012; Trerotola et al., 2013a; Trerotola et al., 2013b; Trerotola et al., 2021). Upregulation of Trop-2 has been associated to poor prognosis of pancreatic, gastric, ovarian, lung and colorectal cancers (Relli et al., 2018; Guerra et al., 2021), consistent with a role in tumor progression (Trerotola et al., 2013a; Trerotola et al., 2013b; Hsu et al., 2020; Trerotola et al., 2021). The Sacituzumab govitecan (TRODELVY, IMMU-132) antibody-drug conjugate (ADC) has been approved by the FDA for therapy of patients with metastatic estrogen receptor-positive and triple-negative breast cancer (Bardia et al., 2019; Bardia et al., 2021) and urothelial carcinomas (Tagawa et al., 2021). However, its half-life is 11–14 h in plasma (Ocean et al., 2017), and causes side effects, such as neutropenia and diarrhea, which are due to rapid release of the SN38 payload (Okajima et al., 2021). These findings indicate an urgent need for next-generation anti-Trop-2-targeted therapy.

Our findings indicated that proteolytic activation of Trop-2 by ADAM10 underlies Trop-2 capacity to drive colon cancer malignant progression (Trerotola et al., 2021). We then showed that Trop-2 proteolytic activation is a pivotal step for acquisition of growth and of metastatic capacity, through cleavage of E-cadherin and inactivation of cell-cell adhesion (Guerra et al., 2021). Trop-2 cleavage does not occur in normal tissues (Guerra et al., 2021; Trerotola et al., 2021), suggesting a unique cancer vulnerability in patients. We exploited this vulnerability through the generation of the 2G10 family of anti-Trop-2 mAb (Guerra et al., 2023a). We then tackled the recognition of Trop-2 within difficult-to-reach, densely-packed tumor sites. The 2EF mAb was developed to favor access to Trop-2 at cell-cell junctions, which are otherwise inaccessible to benchmark anti-Trop-2 antibodies (Guerra et al., 2023b). Synergy between 2EF and 2G10 against tumor xenotransplants was then shown, opening novel avenues for Trop-2-targeted therapy.

Reliable models for *in vivo* toxicity of next-generation therapeutics are urgently needed. These were investigated through exploration of phylogenetic conservation of Trop-2 sequences across vertebrates, then in non-human primates (NHPs). Sequence divergence and incomplete conservation of expression patterns were observed in mouse and rat. Immunohistochemistry analysis of primate tissues showed Trop-2 expression patterns that overlapped with rodent distributions and closely followed those in human tissues. NHP *TACSTD2/TROP2* cDNAs were cloned and transfectants for individual ORF were shown to be efficiently recognized by humanized anti-Trop-2 mAbs, validating NHPs as reliable models for *in vivo* toxicity and pharmacokinetic (PK) profiling. We observed lack of toxicity and prolonged serum half-life of Hu2G10 and Hu2EF in cynomolgus monkey, thus supporting the use of these mAbs as next-generation anti-Trop-2 immunotherapies.

## 2 Materials and methods

### 2.1 DNA transfection

Cells were transfected with DNA (Alberti et al., 1994) in Lipofectamine 2000 or LTX (Invitrogen, Waltham, MA, United States) following manufacturer instructions. Stable transfectants were selected in G-418-containing medium.

### 2.2 Non-human primate tissue samples

Formalin-fixed, paraffin-embedded and flash-frozen tissue samples from rhesus monkey were provided by the European Primate Network (EUPRIM-Net) Biobank, and were analysed for Trop-2 expression by IHC and Western blotting as described. Tissue samples included: bone marrow, brain, colon, duodenum, esophagus, eye, heart, jejunum, kidney, liver, lung, mammary gland, ovary, pancreas, parotid gland, salivary gland, skin, spleen, stomach, thymus, thyroid, tongue, urinary bladder, uterus.

### 2.3 ELISA assay

ELISA assay plates were coated overnight at 4°C with 100 μL/well of 1 μg/mL recombinant human Trop-2-IgFc chimera protein (rhTROP-2, Cat #650-T2-100, R&D, Minneapolis, MN, United States), in 0.2 M sodium carbonate buffer (pH 9.4). Well surfaces were blocked with 300 µL/well of blocking buffer (2% skim milk in PBS, 0.05% Tween-20), for 30 min RT. The plates were washed twice with wash buffer (PBS, 0.05% Tween-20). Purified antibodies or supernatants were added to the plates at appropriate serial dilutions, from 5 to 10 μg/mL, followed by serial 3-fold dilutions, 100 µL/well. All dilutions were performed in blocking buffer. Antibody-containing plates were incubated for 1 h at RT, then washed 3 times with wash buffer. Antibody binding was revealed with 100 µL/well of a 1:2000 dilution of goat anti-human kappa-HRP (Cat # 2060-05, Southern Biotech, Birmingham, AL, United States) in blocking buffer, incubated for 30 min at RT, and followed by four washes with wash buffer. HRP activity was quantified by adding 100 μL/well ABTS substrate (AMRESCO, Solon, OH), activated with 20 μL 30% H_2_O_2_ per 10 mL ABTS solution. The reaction was stopped with 100 µL/well 2% oxalic acid. Absorbance was read at 405 nm.

### 2.4 Flow cytometry

Cell staining for flow cytometry was performed as described (Dell’Arciprete et al., 1996). Fluorescence analysis and cell sorting were carried out with fluorescence-activated cell sorters (FACStar and Vantage, Becton Dickinson, Sunnyvale, CA), after enrichment for expressing transfectants with MAgnetic Cell Sorting-MACS^®^ (Miltenyi Biotec, Bergisch Gladbach, Germany). To improve the detection of transfectants stained with FITC-mAb, subtraction of cell autofluorescence and displacement of FITC-stained cells in the red channel were performed as described (Alberti et al., 1987; Alberti et al., 1991). All Trop-2 transfectants were selected for expression levels comparable to those of endogenously expressing human cancer cells (Alberti and Herzenberg, 1988; Ripani et al., 1998). mAb were conjugated to Alexa488 (Cat.A-21202 Life Technologies, Carlsbad, CA, United States) for single-step staining.

### 2.5 Western blotting

Western blotting was performed as described (Guerra et al., 2022; Guerra et al., 2023a). Cleared tissue lysates were electrophoresed with SDS-PAGE and transferred to nylon filters. Equal loading across gel lanes was verified with Ponceau-red staining of SDS-PAGE gels. Filters were challenged with AF650 anti-human Trop-2 goat polyclonal antibody (pAb; R&D Systems) and signals were developed by chemiluminescence/radiographic film exposure. Semi-quantitative assessments were as follows: (−) no expression; (+) barely detectable; (++) intermediate; (+++) high; (++++) very high expression.

### 2.6 Immunohistochemistry

Immunohistochemistry (IHC) of normal rodent and primate tissues was performed as previously described (Querzoli et al., 2006; Ambrogi et al., 2014). Briefly, specimens were fixed in phosphate-buffered formalin, pH 7.2, and embedded in paraffin. Five-micron sections were mounted on silanized slides, deparaffinized, and rehydrated through graded alcohols to water. Endogenous peroxidase activity was blocked by incubation with 3% H_2_O_2_ for 5 minutes. Antigen retrieval was performed by microwave treatment at 750 W for 10 min in 1 M urea buffer pH 8.0.

Sections were then incubated for 30 min with the AF650 anti-human Trop-2 or AF1122 anti-murine Trop-2 goat antiserum (R&D Systems). To control for non-specific reactivity, the specific primary antibodies were replaced with non-immune serum or with isotype-matched immunoglobulins (DAKO, Agilent, Santa Clara, CA, United States). Anti-goat (K0679, LSAB kit; DAKO) secondary pAbs were used for signal amplification. Slides were washed in Tris-buffered saline-Tween 20 and incubated for 10 min in 3,3′-diaminobenzidine (DAKO). Counterstaining was performed with hematoxylin. Slides were mounted with Immunomount (Shandon, Thermo Scientific, Waltham, MA, United States).

Trop-2 expression was quantified as percentage of stained cells and as intensity of the staining. A positivity score was determined according to five categories: 0 (0% of positive cells), 1 (<10% of positive cells), 2 (10%–50% of positive cells), 3 (50%–80% of positive cells), 4 (>80% of positive cells). An intensity score classified the average intensity of the positive cells as 1 (weak staining), 2 (moderate staining) or 3 (strong staining).

### 2.7 Phylogenetic conservation analysis of Trop-2 across species

The evolutionary history of the *TACSTD2/TROP2* gene was investigated using the Gene Orthology and Paralogy Prediction Pipeline at https://www.ensembl.org/index.html. This uses the longest translation of each gene from every species in Ensembl to build gene trees that represent the evolutionary history of gene families evolving from a common ancestor. Gene trees are then integrated into species trees to pinpoint speciation and duplication events, leading to orthologues and paralogues of the gene under study.

Sequence multi-alignment was performed with Clustal Omega (http://www.ebi.ac.uk/Tools/msa/clustalo/) and was utilized to define the degree of sequence conservation across vertebrate species.

### 2.8 Primate *TROP2* cDNA cloning and expression

Rat and mouse Trop-2 sequences are not recognized by available anti-Trop-2 mAbs (El Sewedy et al., 1998), nor by Hu2G10, Hu2EF (Guerra et al., 2023a; Guerra et al., 2023b), suggesting that rodents do not adequately model on-target/off-tumor toxicity of anti-Trop-2 mAbs. NHP *TACSTD2/TROP2* mRNA and protein sequences were thus retrieved from http://www.ncbi.nlm.nih.gov/; http://blast.ncbi.nlm.nih.gov/. Protein sequence multialignments were performed with Clustal Omega (http://www.ebi.ac.uk/Tools/msa/clustalo/) for *Pan troglodytes* (chimpanzee), *Pongo abelii* (orangutan), *Nomascus leucogenys* (gibbon), *Papio Anubis* (baboon), *Macaca mulatta/fascicularis* (rhesus/cynomolgus monkey), *Callithrix jacchus* (marmoset). Trop-2 sequences were found to be 95%–100% identical to the human sequence.

Gene synthesis of monkey *TROP2* ORF for cynomolgus monkey, baboon, marmoset was performed by Genscript (Genscript Biotech, Piscataway, NJ, United States). All cDNA were resequenced in house, then subcloned into the pEDFP-AX expression vector, between the HindIII and KpnI sites. The primate Trop-2 expression constructs were transfected transiently in human 293 cells, stably in monkey COS-7 cells, with G418-selection and flow-cytometry sorting of expressing populations.

### 2.9 Homology modeling

Model Building_AlphaFold: Trop-2 target structure model was built using AlphaFold algorithms (Jumper et al., 2021; Tunyasuvunakool et al., 2021). AlphaFold is a computational method that can predict protein structures with atomic accuracy. AlphaFold has demonstrated accuracy levels that are competitive with those of experimental structures, over vast numbers of investigated proteins.

Model Building_training dataset: The target sequence and a Trop-2 template structure file in PDB format (7pee.pdb) (Pavšič, 2021) were aligned. Models were built based on the target-template alignment using ProMod3 (Studer et al., 2021) (https://swissmodel.expasy.org/). Coordinates which are conserved between the target and the template were copied from the template to the model. Insertions and deletions were remodeled using a fragment library. Side chains were then rebuilt. The geometry of the resulting model was regularized by using a force field.

Model Quality Estimation: The global and per-residue model quality was assessed using the QMEAN scoring function (Studer et al., 2021). As Trop-2 can acquire dimeric and tetrameric assembly conformations (Pavšič, 2021; Sun et al., 2021), the quaternary structure annotation of the template was used to model the target structure according to potential oligomeric forms. This method (Bertoni et al., 2017) is based on a supervised machine learning algorithm, Support Vector Machines (SVM), which combines interface conservation, structural clustering, and other template features to provide a quaternary structure quality estimate (QSQE).

Model analysis: Swiss-PdbViewer (www.expasy.ch/swissmod/SWISS-MODEL.html), and PyMol (pymol.org/2/) were utilized for graphic rendering of the 3D structures and model analysis.

Model Building_validation dataset: Homology-modeling procedures were subsequently conducted utilizing the Trop-2 3D structure independently determined by Sun et al. (2021). Structure-quality assessment and comparison of the 3D models versus those obtained utilizing the 7pee.pdb (Pavšič, 2021) were utilized for final validation.

### 2.10 Hu2G10 and Hu2EF toxicity and PK in primates

Nine healthy cynomolgus monkeys, six males and three females, 2.5–3.0 years old, 2.37–3.18 kg weight, were obtained from Laboratory Animal Center of the AMMS, Beijing, China. All monkeys were housed in individual cages and allowed free access to water and food.

On day 0 animals (2 males and one female) were assigned to three experimental groups, each group receiving one of the following treatments: 10 mg/kg Hu2G10, 10 mg/kg Hu2EF, 5 mg/kg Hu2G10 + 5 mg/kg Hu2EF. All animals received the indicated mAb dose in 11 mL 0.9% saline solution, which was delivered intravenously as a single infusion at a dosing speed of 3–5 mL/min.

Body weight: All monkeys were weighed at the study initiation, and then once a week until the end of study. All animals were euthanized on day 28 after antibody infusion for histopathology analysis.

Clinical hematological and biochemical measurements: Clinical hematological and biochemical measurements were performed on day 0, 7, 14 and 28. Hematological parameters included white blood cells (WBC), red blood cell (RBC), hemoglobin (HGB), hematocrit (HCT), mean corpuscular volume (MCV), mean corpuscular hemoglobin (MCH), mean corpuscular hemoglobin concentration (MCHC), red cell distribution width (RDW), platelets (PLT), plateletocrit (PCT), mean platelet volume (MPV), platelet distribution width (PDW), white blood cell differential counts of lymphocytes (LYM), monocytes (MON), neutrophils (NEUT), eosinophils (EOS), and basophils (BAS), and were measured in peripheral blood samples.

Blood serum biochemical parameters included albumin (ALB), total protein (TP), alkaline phosphatase (ALP), blood urea nitrogen (BUN), cholesterol (CHOL), alanine aminotransferase (ALT), glucose (GLU), triglyceride (TG), aspartate aminotransferase (AST), total bilirubin (TB), lactate dehydrogenase (LDH), creatinine kinase (CK), serum creatinine (sCr), and amylase (AMY), and were measured in serum samples.

PK assessment: Whole blood samples were harvested into tubes without anti-coagulant on Day 0 (before infusion and 2 h post-infusion), 7, 14, 21 and 28. After clotting, the samples were centrifuged, and sera were collected and stored at −20°C until ELISA assay measurements.

The serum concentrations of Hu2G10 and Hu2EF were determined using anti-idiotypic antibodies in capture-ELISA assays (Supplementary Methods). Wells of ELISA plates were coated with 100 μL/well of 1 μg/mL murine 1D4-1 anti-Hu2G10 or rat #36 anti-Hu2EF anti-idiotypic mAb in PBS overnight at 4°C. After washing with wash buffer (PBS, 0.05% Tween-20), the ELISA plates were blocked with SuperBlock buffer (Thermo Fisher Scientific). Hu2G10 and Hu2EF mAb standard curves were obtained from 3-fold serially-diluted 1 μg/mL solutions in SuperBlock buffer. Eight concentrations of Hu2G10 or Hu2EF standard (Std 1000 ng, Std 333 ng, Std 111 ng, Std 37 ng, Std 12.35 ng, Std 4.12 ng, Std 1.37 ng, and Std 0.46 ng), 50-fold-diluted serum samples, and blank samples were added to the wells in triplicate (100 µL/well). All dilutions were done in SuperBlock buffer. After incubating the ELISA plates for 1 hour at room temperature and washing with wash buffer, the HRP-conjugated goat anti-human kappa secondary pAb (Life Technologies) was added to each well (100 μL/well of a 1/2,000-dilution). After incubating for 30 min at room temperature and washing with wash buffer, color development was initiated by adding 100 μL/well of 3,3′,5,5′-tetramethylbenzidine (TMB) substrate solution, and stopped with 100 μL/well of 2 M H_2_SO_4_ solution. Absorbance was measured at 450 nm. Average optical density (OD) values were calculated for triplicate wells of standards, blanks, and experimental serum samples. Hu2G10 and Hu2EF standard curves were generated by fitting OD values of standard concentrations. Hu2G10 and Hu2EF concentrations in experimental samples were determined using average OD values for each sample (y value) and the equation generated from the standard curve to solve the x value, which represented the concentration of that sample.

### 2.11 Study approval

Procedures involving animals and their care were conducted in compliance with institutional guidelines, national laws and international protocols (D.L. No.116, G.U., Suppl. 40, 18 February 1992; No. 8, G.U., July 1994; UKCCCR Guidelines for the Welfare of Animals in Experimental Neoplasia; EEC Council Directive 86/609, OJ L 358. 1, 12 December 1987; Guide for the Care and Use of Laboratory Animals, United States National Research Council, 1996), following approval by the Animal Protection Committee of the Beijing Experimental Animal Center (Research Proposal Approval, 30 June 2015).

## 3 Results

### 3.1 Phylogenetic conservation of the *TACSTD2*/*TROP2* gene

A phylogenetic tree representing the evolutionary history of the *TACSTD2/TROP2* gene was generated by the Gene Orthology/Paralogy prediction method pipeline (Supplementary Figure S1). A single intron-containing *TACSTD* gene first appeared in vertebrates, then a retro-position event originated the intronless *TACSTD2*/*TROP2* gene, (Fornaro et al., 1995; El Sewedy et al., 1998; Calabrese et al., 2001; Zanna et al., 2007), giving rise to a two-member family of paralog genes, *TACSTD1/TROP1/EPCAM* and *TACSTD2/TROP2.* This duplication event can be located after the divergence of amniotes and amphibians, but before the divergence of mammals and reptiles. No *TROP* genes were identified in invertebrate organisms (Supplementary Figure S1).

Trop-2 homologous sequences were retrieved for NHPs (*Pan troglodyites*/chimpanzee; *Papio anubis*/baboon; *Macaca mulatta-fascicularis*/rhesus-cynomolgus monkey; *Pongo abelii/*orangutan; *Callithrix jacchus*/marmoset), rodents (*Mus musculus*; *Rattus norvegicus*), cow, dog, horse, opossum (*Monodelphis domestica*), chicken and fishes (*Tetraodon nigroviridis*, *Danio rerio*, *Oreochromis mossambicus*). Protein sequence multialignments showed conservation in primary sequence and conservative amino acid changes across vertebrates (Table 1 and Supplementary Figure S2).

**TABLE 1:** Sequence multialignment of human and NHP Trop-2.

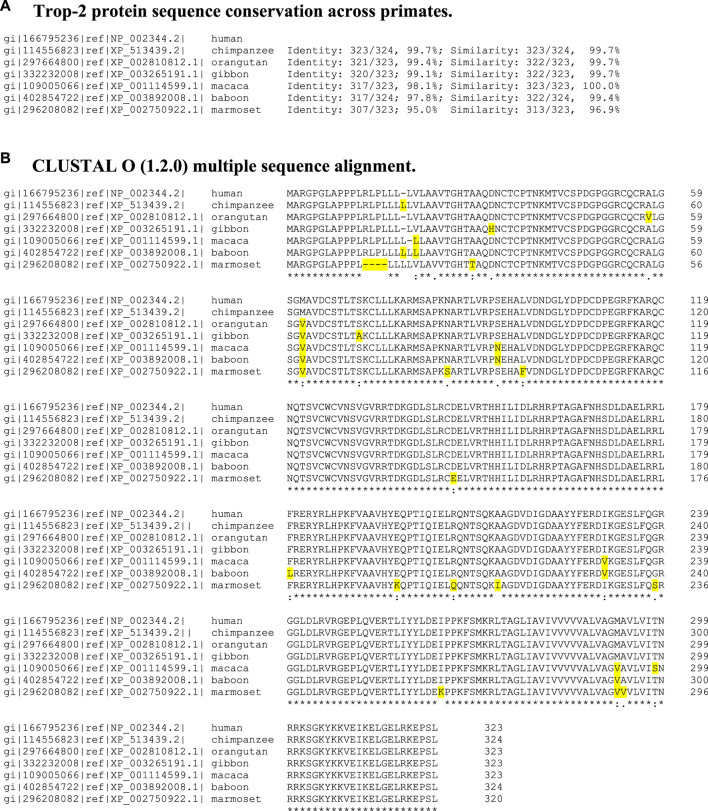

Primate TROP2/TACSTD2-related sequences were retrieved from http://www.ncbi.nlm.nih.gov/; http://blast.ncbi.nlm.nih.gov/. Protein sequence multialignments were performed with Clustal Omega (http://www.ebi.ac.uk/Tools/msa/clustalo/). (A) Percent identity. (B) Sequence multialignment. Yellow highlights: sequence variation/polymorphic residues.

### 3.2 3D structure of primate Trop-2

Model Building_AlphaFold: Trop-2 target structure model was built using AlphaFold algorithms (Jumper et al., 2021; Tunyasuvunakool et al., 2021). AlphaFold is a novel machine learning approach that incorporates physical and biological knowledge about protein structure and utilizes multi-sequence alignments for generating a deep learning algorithm. *Ab initio* AlphaFold prediction of the structure of Trop-2 was performed (Supplementary Table S1). Quality assessment of the resulting model was conducted. Ramachandran plots of F.versus Y. peptide bond angle analysis (Trerotola et al., 2021) in cynomolgus monkey Trop-2 versus human X ray crystal structure-modeled Trop-2 identified 94.89% favored residues versus 0.43% outliers, with a clash score of 0.27 and 11/2,582 bad peptide angles. A corresponding analysis on modeling that utilized a Trop-2 AlphaFold-constructed structure identified 90.03% favored residues versus 2.49% outliers, with a clash score of 0.40 and 17/3,453 bad peptide angles.

Model Building: Primate Trop-2 protein sequence multialignment showed high degree of sequence conservation (Table 1). Only one amino acid difference between chimpanzee and human Trop-2 was detected, as an additional Leu in the chimpanzee leader peptide, making the mature Trop-2 identical to that in human. NHP Trop-2 protein sequences were found to be between 99.4% (orangutan) and 95% (marmoset) identical to the human one. The two rhesus and cynomolgus monkeys (from the macaque family) were found to have identical Trop-2 sequence. Sequence similarity ranged from 100% to 96.9%, indicating conservative amino acid replacement in most instances (Table 1).

These findings indicated the feasibility of 3D structure homology modeling, using the human Trop-2 structure (Pavšič, 2021) as a template. Target sequence and template structure files in PDB format were aligned using ProMod3 algorithms (Studer et al., 2021). Coordinates which were conserved between the target and the template were utilized for the models. Insertions and deletions were remodeled using a fragment library and the geometry of the resulting models was refined using a force field. Model quality was assessed using the QMEAN scoring function (Studer et al., 2021). As Trop-2 can acquire dimeric and tetrameric conformations (Pavšič, 2021; Sun et al., 2021), the quaternary structure annotation of the template was used to refine the model to permit the acquisition of an oligomeric form (Bertoni et al., 2017) (Supplementary Table S1).

**FIGURE 1:**
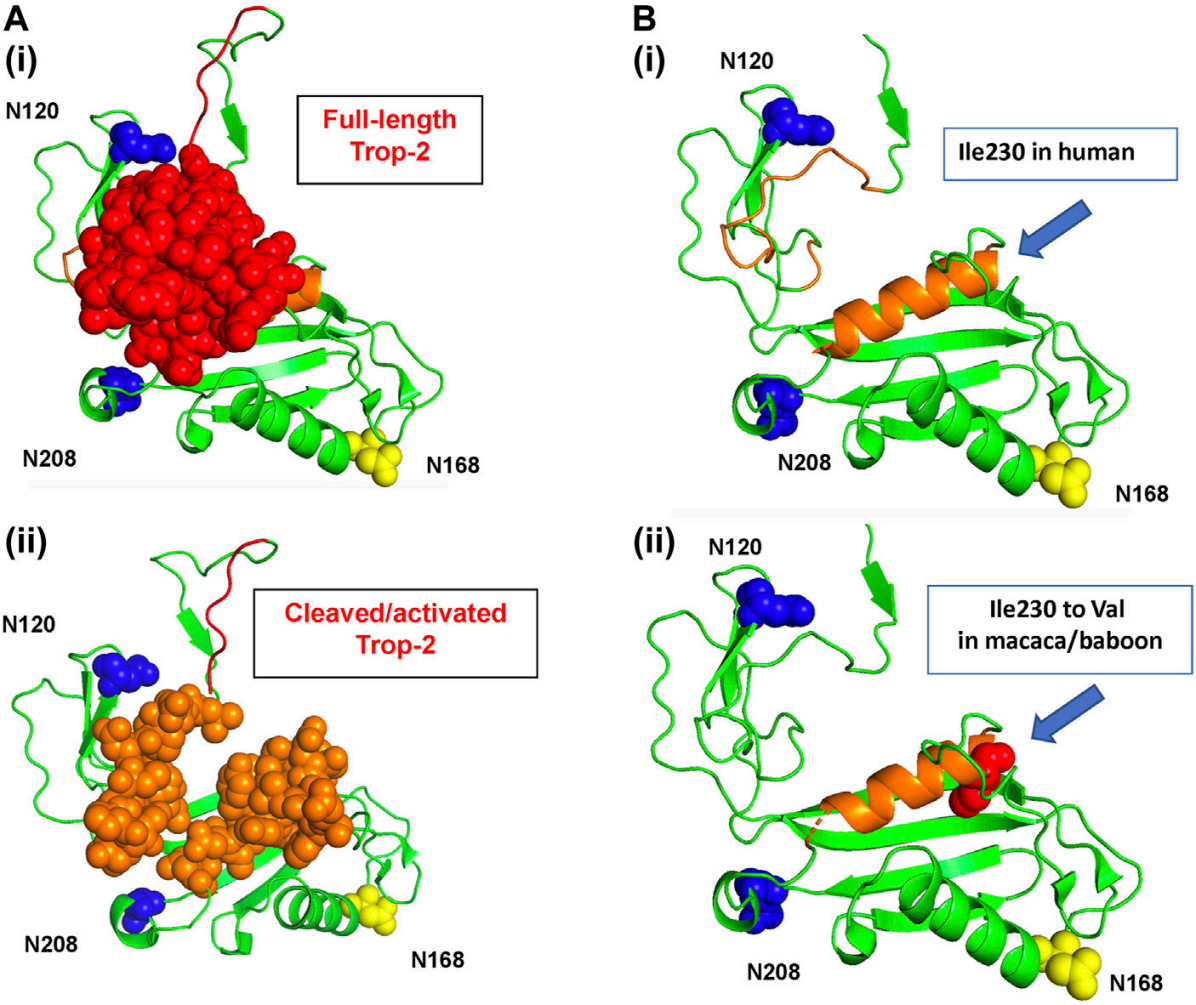
The human Trop-2 protein structure. The 3D structure of the extra-cellular domain of Trop-2 (Pavšič, 2021) is in ribbon diagrams and sphere/protein surface models. Trop-2 N-glycosylation sites are indicated. N120 and N-208 are in blue; the N120A and N-208A mutants were shown to abolish binding of Claudin-7 to Trop-2 (Mori et al., 2019; Kamble et al., 2021). The N168 glycosylation site is in yellow. **(A)** Top view. (i) The N-terminal subunit of Trop-2 is in red as sphere model; (ii) the 3D structure of the extra-cellular domain of Trop-2 devoid of the N-terminal subunit is shown. The groove between the glycans at N120 and N208 that becomes more accessible upon ADAM10-cleavage and rearrangement of the N-terminal ADAM10-cleaved subunit (Guerra et al., 2023a) is in orange, sphere model. **(B)** Ribbon diagrams of the activation site of Trop-2 (Mori et al., 2019; Kamble et al., 2021), and binding site of Hu2G10 (Guerra et al., 2023a) are provided for clarity. Blue arrow: polymorphic residue across NHP species. (i) Human Ile230 residue. (ii) Space-fill model of cynomolgus and baboon Val230 is provided. The residue is buried at the bottom of the orange α-helix, below the solvent-exposed region.

**FIGURE 2:**
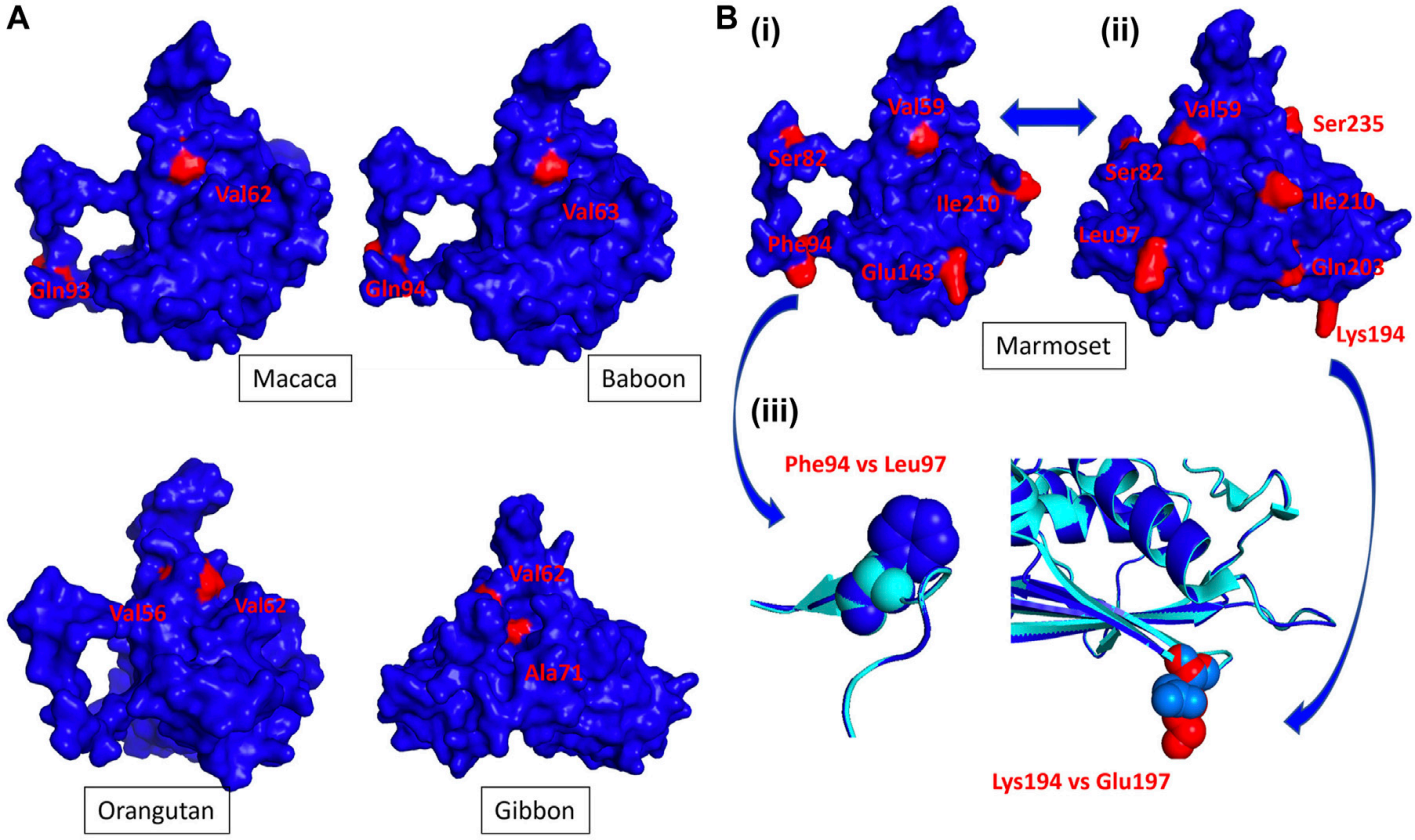
The NHP Trop-2 protein structure. NHP 3D structures were modeled versus the crystal structure of the human Trop-2 (Pavšič, 2021), following the described procedures (Materials and Methods). The 3D modeling parameters are listed in Supplementary Table S1. Polymorphic residues versus the human Trop-2 sequence are in red. **(A)** Side (macaca, baboon, orangutan) and frontal (gibbon) views of primate Trop-2s (Supplementary Table S1). The 3D model surface representation was obtained with PyMol (Supplementary Table S1). **(B)** (i) Side and (ii) frontal views of the marmoset Trop-2 3D structure. (iii) Overlap of the human and marmoset Trop-2 is in ribbon/contour diagrams. Alpha helices, beta sheets (flat arrows) and loops are shown. The ribbon diagram of marmoset Trop-2 is in blue; that of the human Trop-2 is in cyan. (*blue arrows*) Phe94 in marmoset versus Leu97 in human and Lys194 in marmoset versus Glu197 in human are magnified.

The Trop-2 models revealed a highly conserved geometry (Supplementary Table S1; Figures 1, 2). Overlap assessment of target sequences and human Trop-2 revealed parallel folding modes also versus the most distant sequences, e.g., marmoset (Supplementary Table S1, cartoon structures). The groove between the glycans at N120 and N208, which becomes accessible upon ADAM10-cleavage (Guerra et al., 2023a) was explored. This elongated groove contains the D218-K231 α-helix, and the three G232-Q237, G102-F114 and V131-G132 loops. Ile230 was found to be the only polymorphic residue in this region across primate species, Ile230Val occurring in rhesus/cynomolgus monkey and baboon (Table 1). This residue was found buried at the bottom of the groove and shielded from solvent by the D218-K231 α-helix (Figure 1).

Homology-modeling procedures were subsequently conducted utilizing the Trop-2 3D structure independently determined by Sun et al. (Sun et al., 2021). Comparison of the 3D models obtained utilizing 7e5n.1.pdb versus 7pee.pdb (Pavšič, 2021) were performed. Quality assessment parameters of the two sets of 3D structures were shown to be essentially identical (Supplementary Table S1F), providing formal validation for our approach.

Analysis of the additional polymorphic residues in NHPs showed limited access to the surface of the Trop-2 structure, suggesting highly conserved surfaces of interaction with other binding partners and supporting the potential for efficient cross-recognition by anti-human Trop-2 antibodies. Twelve residues of Trop-2 were found polymorphic versus the human counterpart only in marmoset, eight of twelve residues being exposed at the surface (Figure 2B). Most divergent among them were Lys94 versus Glu197 in man, and Phe94 versus Leu97 in man. This suggested divergence of surface structure and charge between marmoset and human Trop-2, and proposed this coding sequence as a key discriminant for anti-Trop-2 mAb recognition. Hence, we proceeded on with the cloning of the marmoset *TROP2* and of cognate NHP sequences.

### 3.3 Primate *TROP2* cDNA cloning and expression

*TROP2* ORF were synthesized for cynomolgus monkey, baboon, marmoset. Transient transfectants of marmoset Trop-2 expression constructs were generated in HEK-293 cells. Human, baboon, cynomolgus monkey *TROP2* cDNA were stably transfected in COS-7 cells, selected with G418 and sorted by flow cytometry using the AF650 anti-Trop-2 goat pAb for average cancer cell levels of expression.

**FIGURE 3:**
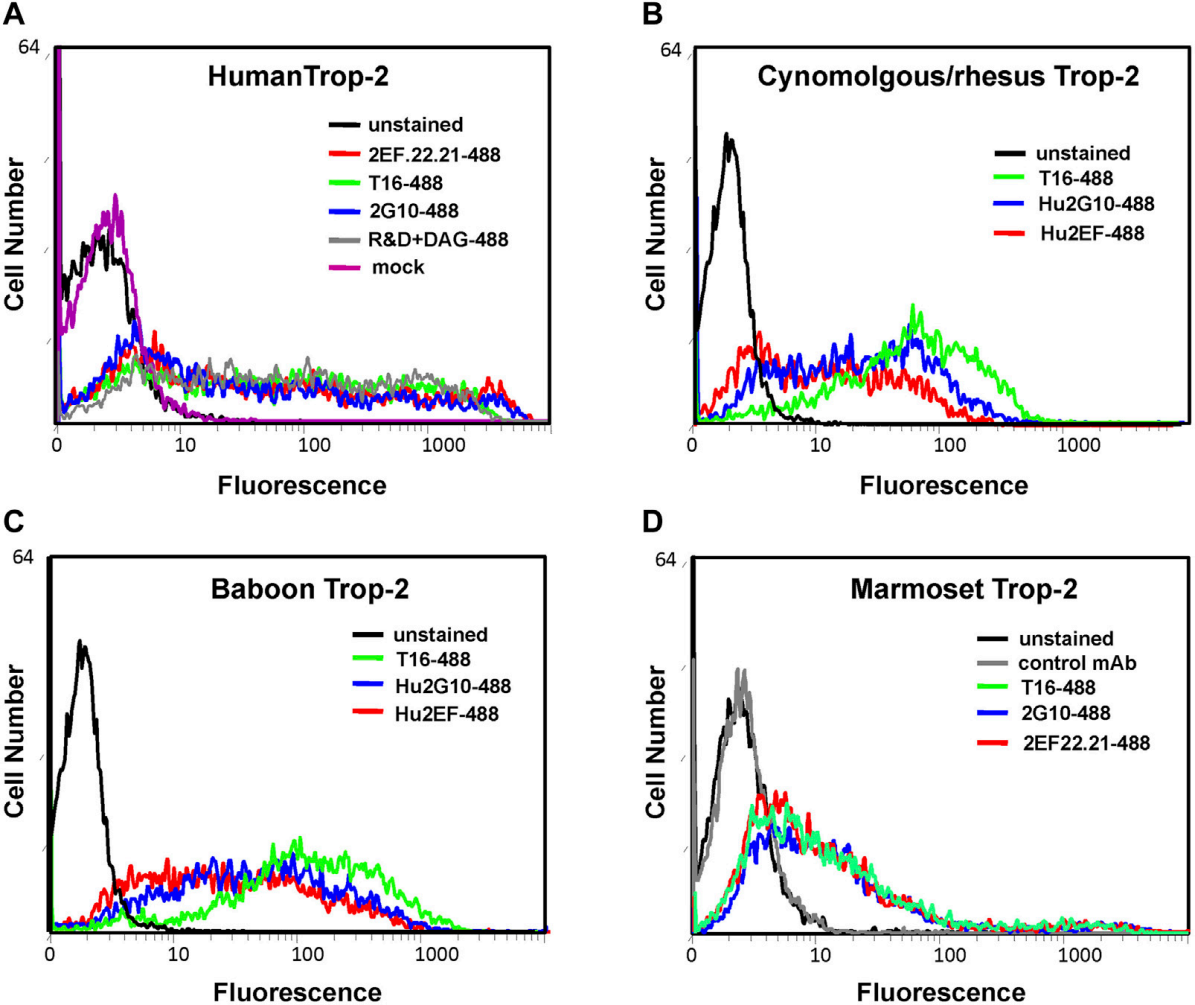
Flow cytometry analysis of NHP cDNA transfectants with the indicated anti-Trop-2 mAbs. Transient transfectants of marmoset *TROP2* cDNA were generated in HEK-293 cells. Human, baboon and rhesus/cynomolgus monkey *TROP2* cDNA were stably transfected in COS-7 cells, following G418-selection and flow-cytometry sorting of expressing population. **(A)** Cells transfected with the human Trop-2 cDNA. **(B)** Cells transfected with the rhesus/cynomolgus monkey Trop-2 cDNA **(C)** Cells transfected with the baboon Trop-2 cDNA. **(D)** HEK-293 cells transiently transfected with the marmoset Trop-2 cDNA. Efficient binding of 2EF/Hu2EF-Alexa488 (red profiles) and 2G10/Hu2G10-Alexa488 (blue profiles) was found to all NHP Trop-2 transfectants. The Alexa488-tagged AbT16 anti-Trop-2 mAb (green profiles) was used as benchmark. **(D)** An irrelevant mAb was used as negative control (gray profile); **(A)** staining using the AF650 anti-Trop-2 goat pAb was used as a positive control (gray profile). Mock transfected cells are in magenta. Unstained cells are in black. Autofluorescence compensation was used in all analyses (Alberti et al., 1987).

Flow cytometry analysis of transfectants was then performed for recognition of NHP Trop-2 by directly fluorochrome-conjugated 2G10/Hu2G10 and 2EF/Hu2EF. Trop-2 was efficiently recognized by the 2G10 and the 2EF mAb families in all tested NHP Trop-2 transfectants. Lower absolute intensity of binding was observed for marmoset Trop-2 (Figure 3).

### 3.4 IHC analysis of Trop-2 expression in NHP tissues

**FIGURE 4:**
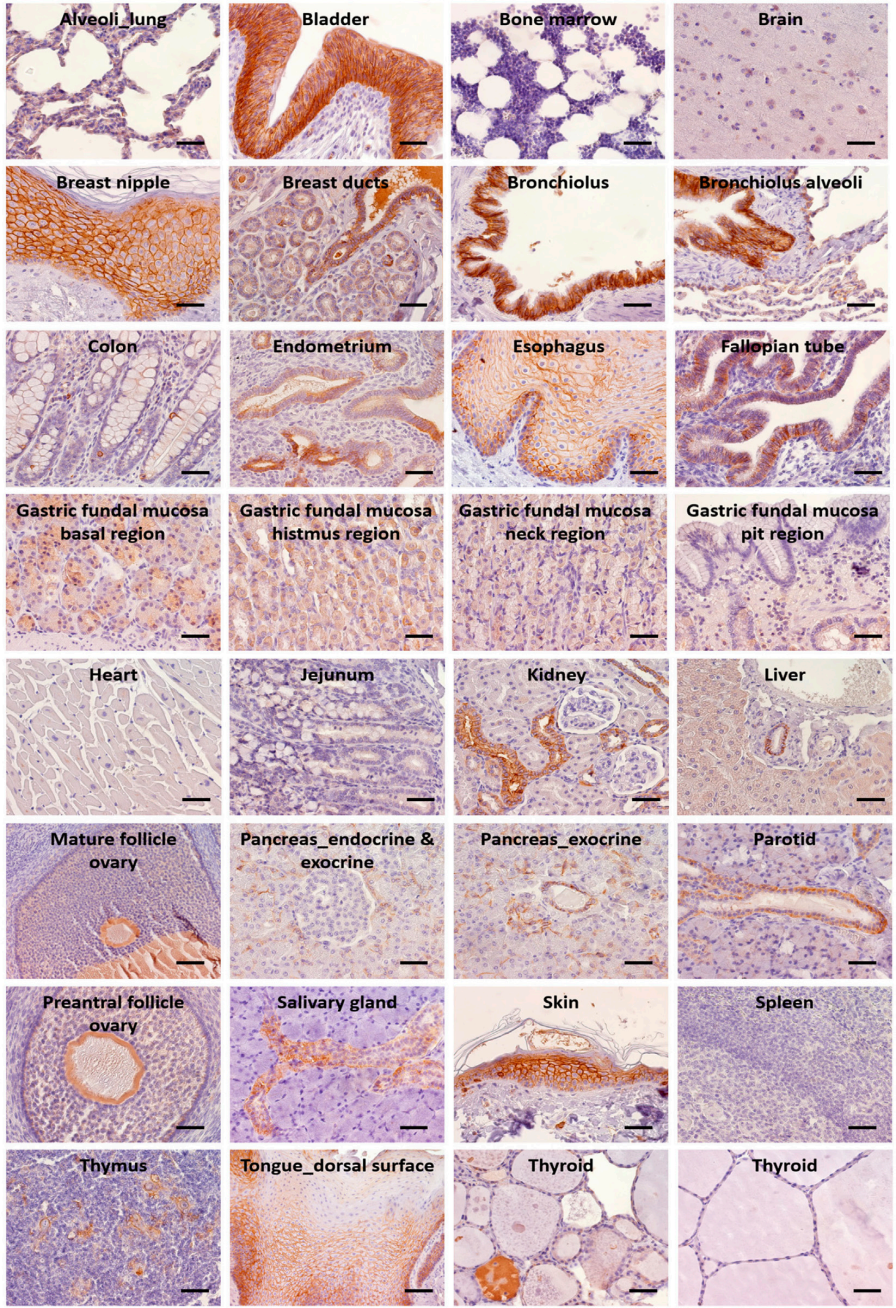
IHC analysis of Trop-2 protein expression in rhesus monkey tissues. Staining was performed with the AF650 anti-Trop-2 goat pAb. Individual organs are indicated. Brown staining reveals Trop-2 expression. Trop-2 expression was quantified as percentage of stained cells and as intensity of the staining. An IHC positivity score was determined according to five categories: 0 (0% of positive cells), 1 (<10% of positive cells), 2 (10%–50% of positive cells), 3 (50%–80% of positive cells), 4 (>80% of positive cells). An intensity score classified the average intensity of the positive cells as 1 (weak staining), 2 (moderate staining) or 3 (strong staining) (Supplementary Table S2). Bars: 50 µm.

IHC determinations in NHPs showed tissue reactivity for Trop-2 that closely paralleled that of human tissues (Supplementary Table S2; Figure 4). High levels of Trop-2 were detected in multi-stratified epithelia of esophagus, tongue, skin, together with hair follicles and breast nipple. Medium to high expression was observed in uterine tubes, endocervix and exocervix, epididymis, urothelium, prostate. Weak expression was detected in liver, gallbladder, thyroid, ovarian follicles, Fallopian tubes.

Compartment-specific expression was found in stomach (base, neck, isthmus), small intestine and colon (crypts), lung (alveolar cells, bronchial epithelial cells), salivary glands (mucous and myoepithelial cells were Trop-2 negative; the salivary ducts expressed low to high levels of Trop-2 in the intra- and inter-lobular ducts, respectively), thymus (Hassall’s corpuscles). In the kidney, distal tubules were positive, whereas no expression of Trop-2 was present in the glomerulum, proximal tubules and the collecting ducts. Pancreas acinar cells and ductal cells were Trop-2 positive, whereas the islets of Langerhans were negative. Endometrial glands were Trop-2 positive.

Spleen, seminiferous tubules, striated muscle cells of the skeletal system and the myocardium, bone marrow, and the endocrine organs—e.g., thyroid follicular epithelium and adrenal cortex—were Trop-2 negative.

More in detail, the stratified squamous epithelium lining the esophagus and tongue showed no Trop-2 expression in the basal cells layer while a strong membrane immunoreactivity was present in the upper layers. Consistent with this gradient pattern, the keratinised squamous epithelium of the epidermis displayed increasing Trop-2 expression from the near-negative basal cell layer to the strongly Trop-2 positive stratum granulosum. Intense Trop-2 staining was also detected in the hair follicles. The transitional epithelium covering the efferent urinary tract showed strong Trop-2 positivity in the basal and intermediate layers, while the umbrella cells were Trop-2 negative. The columnar epithelium lining the oviducts as well as the epithelium of the endocervix and the squamous epithelium of the exocervix all showed strong Trop-2 immunoreactivity. In the male genital tract Trop-2 expression was present in the epididymis, with predominant localization at the lateral membrane, but not in the seminiferous tubules and interstitium.

The highest levels of Trop-2 were detected at the cell membrane. A weaker Trop-2 intracellular staining was revealed. This was due to synthesis in the ER and transport through the Golgi to the cell membrane via intracellular vesicles (Ambrogi et al., 2014). Additional Trop-2 intracellular staining was likely due to storage vesicles, as generated by internalization from the cell membrane (Guerra et al., 2022; Guerra et al., 2023a). Neuronal cells showed Trop-2 cytoplasm immunoreactivity with low/nil membrane staining. A similar pattern of reactivity was found in jejunum crypts, hepatocytes and ovarian follicles (Supplementary Table S2). The terminal duct lobular unit of mammary glands showed Trop-2 cytoplasm immunoreactivity with low/nil membrane staining, overall suggesting a differential regulation of Trop-2 transport in different tissues.

Staining patterns in rodents were found similar, though apparently with a more limited organ expression than in NHPs (full description is provided in Supplementary Results). Corresponding patterns to human samples/high expression levels were observed in rodent skin, hair follicles, tongue, esophagus, pancreas. Species-specific expression was detected in the murine mammary glands and kidney’s distal convoluted tubules and in the rat sublingual salivary gland ducts under the form of cytoplasmic localization (Supplementary Results; Supplementary Figures S3, S4; Supplementary Table S2).

### 3.5 Western blot analysis

**FIGURE 5:**
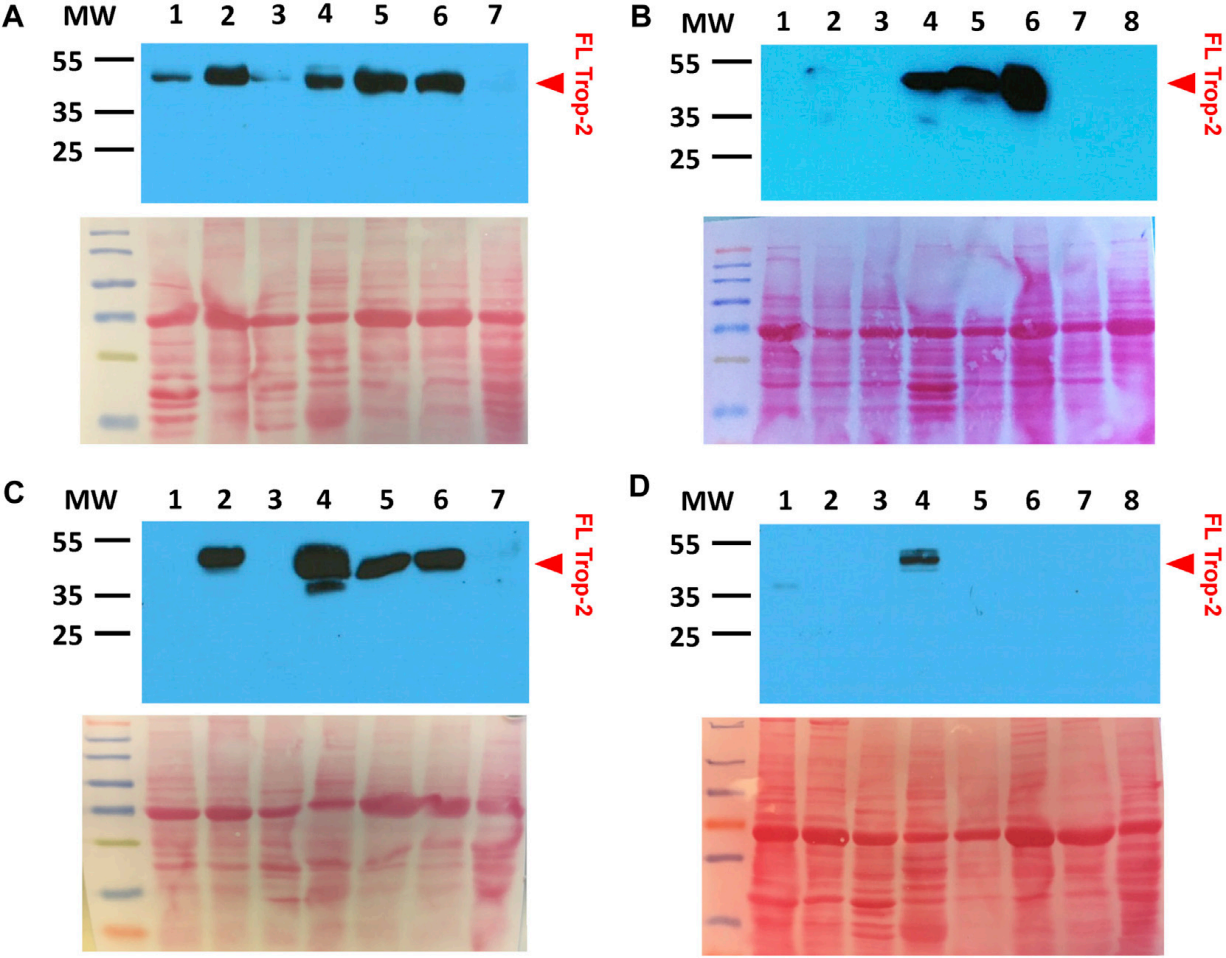
Lack of Trop-2 cleavage in rhesus monkey normal tissues. **(A)** 1: tongue; 2: urinary bladder; 3: heart; 4: salivary gland; 5: mammary gland; 6: skin; 7: kidney. **(B)** 1: brain; 2: eye; 3: thyroid; 4: parotid gland; 5: esophagus; 6: lung; 7: liver; 8: pancreas. **(C)** 1: uterus; 2: urinary bladder; 3: heart; 4: salivary gland; 5: mammary gland; 6: skin; 7: kidney. **(D)** 1: stomach; 2: spleen; 3: thymus; 4: tongue; 5: duodenum; 6: colon; 7: jejunum; 8: ovary. Western blotting was performed with the AF650 anti-Trop-2 goat pAb. Ponceau-red stained SDS-PAGE gels are shown below each Western blot for assessing equal lane loading. MW: molecular weight markers. Prestained mw markers were utilized for clarity. Red arrowhead, FL: full length Trop-2.

Western blot analysis was performed with the AF650 anti-Trop-2 goat pAb, which is directed against the Trop-2 extra-cellular domain. This revealed Trop-2 expression in: (−/+) heart, brain, eye, thyroid, kidney, pancreas, liver, uterus, ovary, spleen, thymus, stomach, duodenum, jejunum, colon; (++) tongue, urinary bladder, parotid gland/salivary glands, mammary gland; (+++/++++) lungs, skin, esophagus (Figure 5). These findings were consistent with the IHC data, but proved less sensitive to Trop-2 expression in small cell subpopulations in target organs, that were more easily revealed by IHC.

Consistent with evidence from human tissues, analysis of normal rhesus monkey organs indicated essentially no cleavage of Trop-2, as derived from the occurrence of ADAM10 cleavage at the first loop of the Trop-2 thyroglobulin domain (Guerra et al., 2021; Trerotola et al., 2021) (Figure 5). This suggested the macaque as a relevant experimental model, where to test Hu2G10 and Hu2EF for potential toxicity *in vivo,* as a proxy for clinical studies in patients.

### 3.6 Hu2G10 and Hu2EF PK in NHPs

A 28-day PK study was carried out in groups of three cynomolgus monkeys. Serum concentrations of Hu2G10 and Hu2EF were measured at day 0 (before infusion and 2 h post-infusion), and weekly after the infusion. using the anti-idiotypic 1D4-1 and #36 mAb, respectively (Supplementary Figure S5; Supplementary Table S3).

**FIGURE 6:**
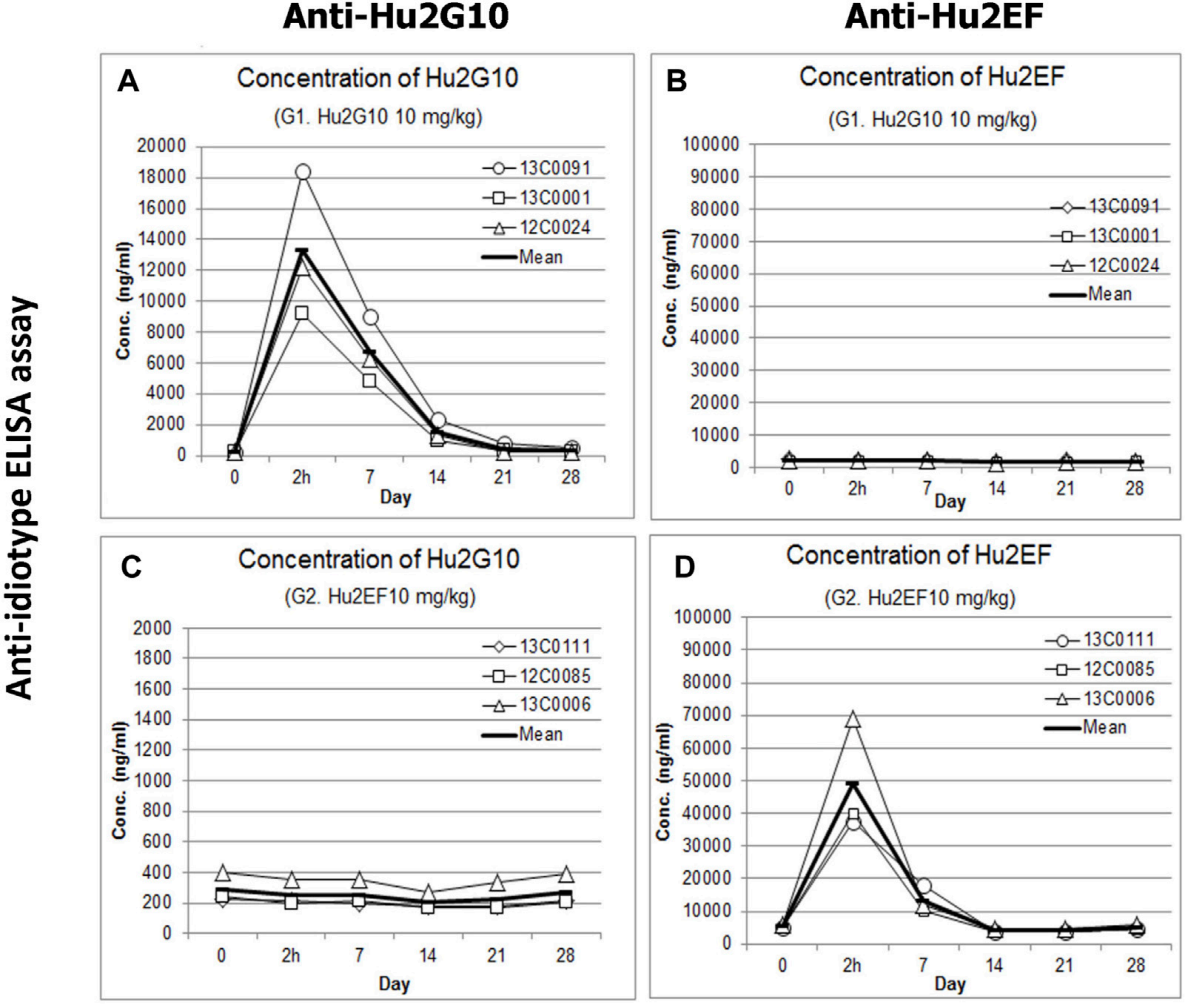
Pharmacokinetics of Hu2G10 and Hu2EF in cynomolgus monkey. Three individual cynomolgus monkeys for each experimental group received an intravenous infusion of 10 mg/Kg of Hu2G10 or 10 mg/Kg of Hu2EF or 5 mg/Kg of Hu2G10 plus 5 mg/Kg of Hu2EF at day 0. Blood serum concentrations of Hu2G10 and Hu2EF were measured by ELISA capture assays with anti-idiotypic antibodies (Supplementary Materials and Methods) at the indicated time points, over 28 days after mAb injection. Absolute parameter values are reported in Supplementary Table S3. The curves displaying average determinations (mean values of replica wells) are in bold. Standard curves of Hu2G10 and Hu2EF progressive dilutions, as included in individual ELISA assay plates, were used as reference (Supplementary Table S3). **(A, B)** Blood serum concentrations of Hu2G10 and Hu2EF, respectively, in the experimental group receiving 10 mg/kg of Hu2G10. **(C, D)** Blood serum concentrations of Hu2G10 and Hu2EF, respectively, in the experimental group receiving 10 mg/kg of Hu2EF.

Before the infusion, background reactivities were undetectable in all serum samples. Hu2G10 serum concentration rapidly increased after intravenous infusion of 10 mg/kg, and reached a peak 2 h after the injection (Supplementary Table S3), with a mean value of 13280.7 ng/mL ± 4,686.3 ng/mL. The Hu2G10 concentration then gradually decreased and reached baseline levels on day 21 (Figure 6). When administered at 5 mg/kg, in combination with Hu2EF, the Hu2G10 serum concentration peak was lower (5759.5 ng/mL ± 1728.7 ng/mL), and circulating Hu2G10 reached baseline levels on day 14 (Supplementary Table S3). Thus, Hu2G10 is stable in plasma and is detectable in the circulation up to 3 weeks after the infusion (t_1/2_ = 6.5 days), in a dose-dependent manner.

Hu2EF serum concentration rapidly increased after intravenous infusion of 10 mg/kg, and reached a peak 2 h after the injection (48921.6 ng/mL ± 17419.5 ng/mL) (Supplementary Table S3). The Hu2EF concentration then gradually decreased and reached baseline levels on day 14 (Figure 6). When administered at 5 mg/kg, in combination with Hu2G10, the Hu2EF serum concentration peak was lower (33385.4 ng/mL ± 184.1 ng/mL) and reached baseline levels on day 14 (Supplementary Table S3). Thus, Hu2EF is stable in plasma, and is detectable in the circulation up to two and a half weeks after the infusion (t_1/2_ = 5.5 days), in a dose-dependent manner.

### 3.7 Hu2G10 and Hu2EF safety in NHPs

All the cynomolgus monkeys in the three experimental groups remained in good health. Hu2G10 and Hu2EF were well tolerated at doses of 10 mg/kg or 5 mg/kg in all monkeys. Neither significant neurological, respiratory, digestive and urinary symptoms, including vomiting, diarrhea, anorexia, nor biochemical and hematological toxicities were found during the 28-day observation. Consistent with this, no body-weight loss was observed in any of the monkeys (Supplementary Table S3).

**FIGURE 7:**
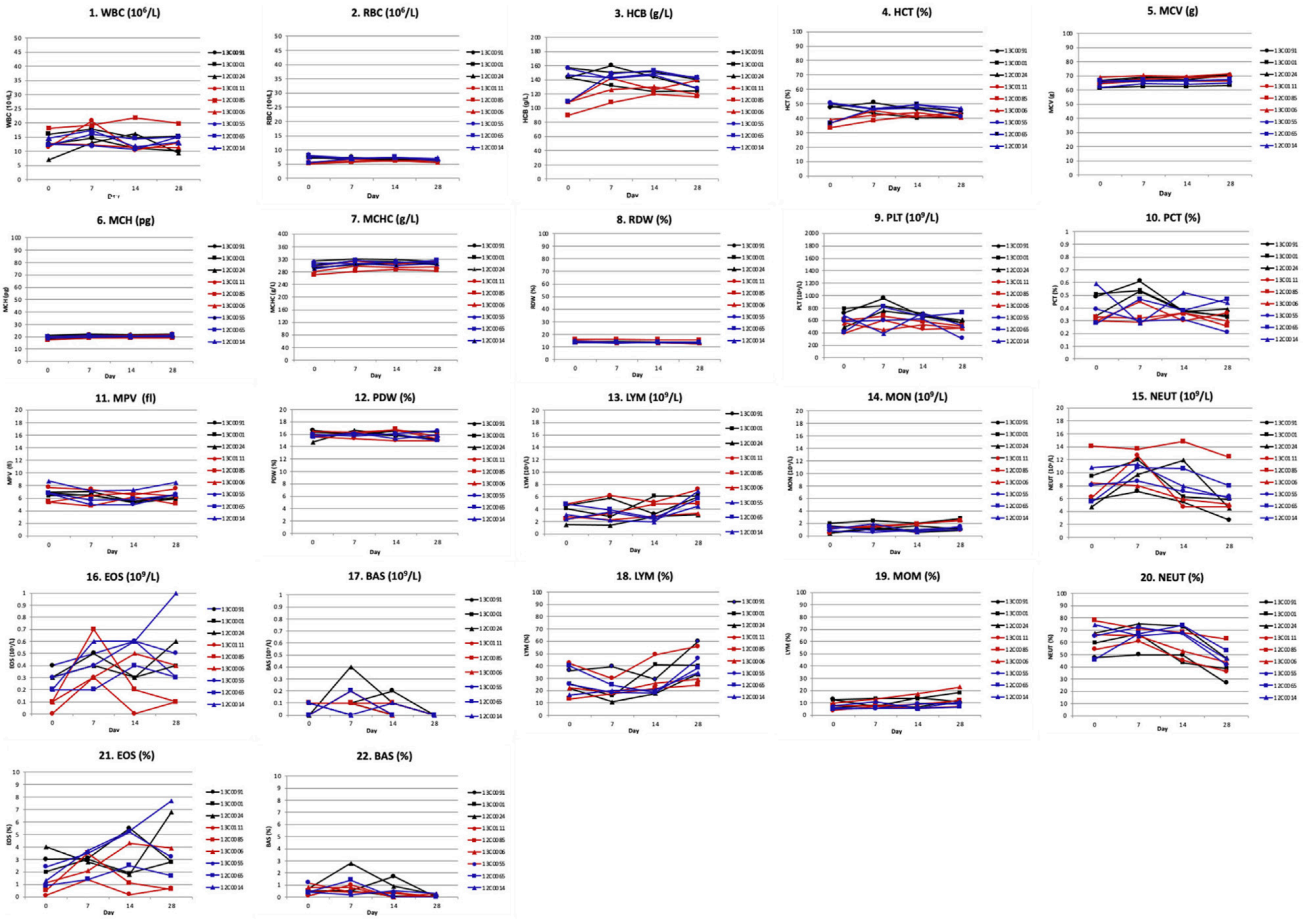
Hematological toxicity of Hu2G10 and Hu2EF in cynomolgus monkey. Hematological determinations were performed on day 0, 7, 14 and 28 after mAb infusion. Hematological parameters included white blood cells (WBC), red blood cells (RBC), hemoglobin (HGB), hematocrit (HCT), mean corpuscular volume (MCV), mean corpuscular hemoglobin (MCH), mean corpuscular hemoglobin concentration (MCHC), red cell distribution width (RDW), platelets (PLT), plateletocrit (PCT), mean platelet volume (MPV), platelet distribution width (PDW), white blood cell differential counts of lymphocytes (LYM), monocytes (MON), neutrophils (NEUT), eosinophils (EOS), and basophils (BAS). The black curves correspond to the monkeys receiving 10 mg/Kg of Hu2G10, the red curves correspond to the monkeys receiving 10 mg/Kg of Hu2EF, the blue curves correspond to the monkeys receiving 5 mg/Kg of Hu2G10 plus 5 mg/Kg of Hu2EF. Absolute parameter values are reported in Supplementary Table S3. Differential counts for monocytes, neutrophils and basophils showed essentially no change during the 28-day observation period, except for a minor increase in percent counts of lymphocytes and eosinophils in some monkeys. No significant differences in WBC counts were recorded before and after infusion in any monkey group.

White blood cell counts: Hematological parameters included white blood cells (WBC), red blood cells (RBC), hemoglobin (HGB), hematocrit (HCT), mean corpuscular volume (MCV), mean corpuscular hemoglobin (MCH), mean corpuscular hemoglobin concentration (MCHC), red cell distribution width (RDW), platelets (PLT), plateletocrit (PCT), mean platelet volume (MPV), platelet distribution width (PDW), white blood cell differential counts of lymphocytes (LYM), monocytes (MON), neutrophils (NEUT), eosinophils (EOS), and basophils (BAS). WBC differential counts for monocytes, neutrophils and basophils showed essentially no change during the 28-day observation period, except for a minor increase in percent counts of lymphocytes and eosinophils in some monkeys. No significant differences in WBC counts were recorded before and after infusion in any monkey group (Supplementary Table S3; Figure 7).

**FIGURE 8:**
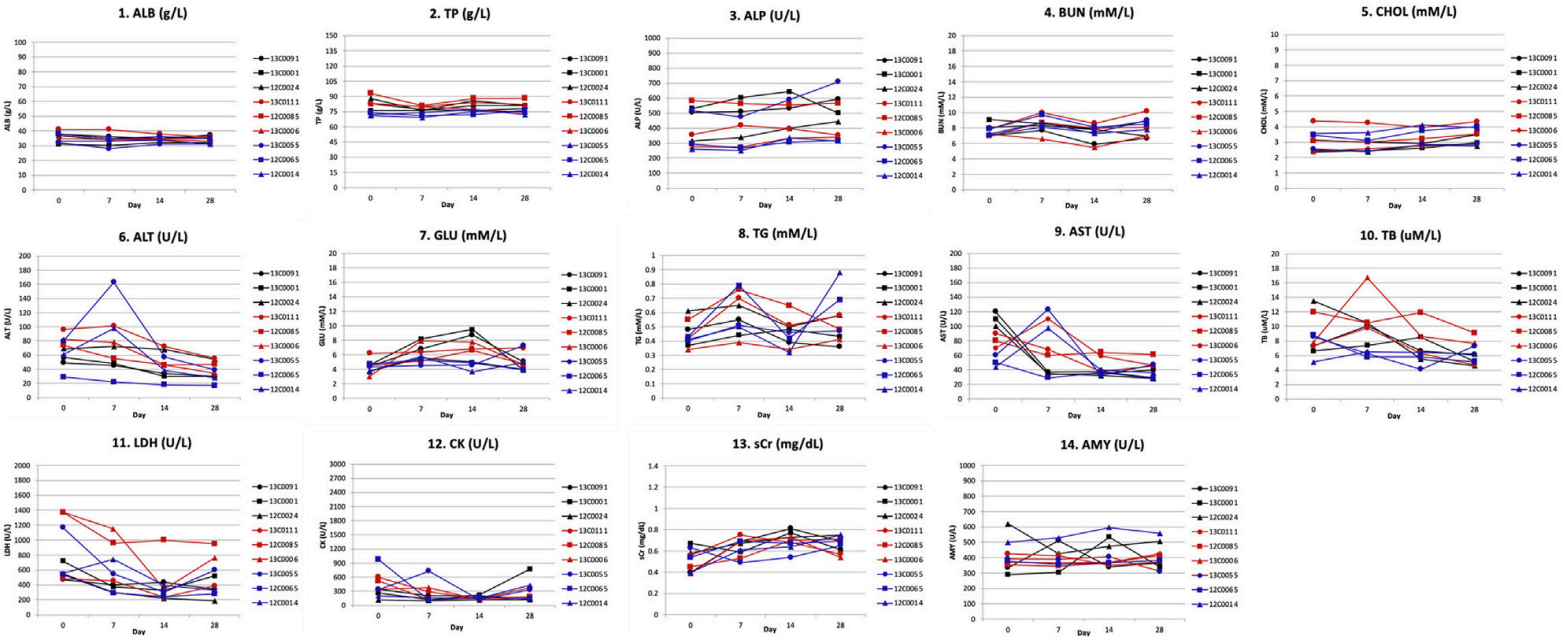
Blood biochemistry toxicity parameters after Hu2G10 and Hu2EF administration to cynomolgus monkey. Biochemical determinations were made in blood serum samples on day 0, 7, 14 and 28 after mAb infusion. Serum biochemical parameters included albumin (ALB), total proteins (TP), alkaline phosphatase (ALP), blood urea nitrogen (BUN), cholesterol (CHOL), alanine aminotransferase (ALT), glucose (GLU), triglyceride (TG), aspartate aminotransferase (AST), total bilirubin (TB), lactate dehydrogenase (LDH), creatinine kinase (CK), serum creatinine (sCr), and amylase (AMY). The black curves correspond to the monkeys receiving 10 mg/Kg of Hu2G10, the red curves correspond to the monkeys receiving 10 mg/Kg of Hu2EF, the blue curves correspond to the monkeys receiving 5 mg/Kg of Hu2G10 plus 5 mg/Kg of Hu2EF. Absolute parameter values are reported in Supplementary Table S3. No significant differences in biochemical parameter values were recorded before and after infusion in any monkey group.

Serum biochemistry determinations: Serum biochemical analyses did not show significant changes in albumin (ALB), total protein (TP), alkaline phosphatase (ALP), blood urea nitrogen (BUN), cholesterol (CHOL), alanine aminotransferase (ALT), glucose (GLU), triglyceride (TG), aspartate aminotransferase (AST), total bilirubin (TB), lactate dehydrogenase (LDH), creatinine kinase (CK), serum creatinine (sCr), and amylase (AMY) levels in any of the monkeys during the 28-day observation (Supplementary Table S3; Figure 8).

## 4 Discussion

We conducted a phylogenetic conservation analysis of Trop-2 across species in vertebrates. The *TACSTD2/TROP2*, as a retrotransposon of *TACSTD1/EPCAM*, was found to first appear in evolution after the branching of tetrapods into amniotes and amphibians, but before the divergence of mammals and reptiles. Hence a single intron-containing *TROP* gene is found in fishes, while the intronless *TROP2* can be found in reptiles, birds and mammals. The Trop-2 proteins were found to share a high degree of sequence homology across species, consistent with a strongly conserved physiological role in higher organisms. This allowed to explore rodent and primate genomics in pre-clinical benchmarks model anti-Trop-2 therapy. Sequence divergence and incomplete conservation of expression patterns were observed in mouse and rat. The highest level of similarity was found between human and NHP Trop-2. The mature chimpanzee and human Trop-2 were found to be identical. The Trop-2 protein sequences in more distant NHP were found to be between 99.4% (orangutan) and 95% (marmoset) identical to the human, with sequence similarity from 100% to 96.9%, suggesting conservation in structure and function. Corresponding findings were obtained through IHC analysis of NHP versus human tissues.

Consistent with this, the primate Trop-2 3D models were found to share a highly conserved geometry and parallel folding modes also versus the most distant sequences, e.g., marmoset. The groove between the glycans at N120 and N208, which becomes accessible upon ADAM10-cleavage and rearrangement of the N-terminal ADAM10-cleaved subunit (Guerra et al., 2023a), acts as the activation site of Trop-2 (Mori et al., 2019; Kamble et al., 2021), and is the binding site of Hu2G10 (Guerra et al., 2023a). The groove geometry was found conserved in all NHP Trop-2. Ile230 was found to be the only polymorphic residue in this region across NHP species, Ile230Val appeared buried at the bottom of the groove and shielded from external contacts in the Trop-2 structure. Additional polymorphic residues in NHPs showed limited overall access to the surface of the Trop-2 structure, consistent with a conserved surface of the Trop-2 molecule and potential cross-recognition by anti human-Trop-2 antibodies. The most relevant exception was the marmoset Trop-2, whereby twelve residues were found polymorphic and largely exposed at the surface of the molecule, suggesting divergence of surface structure and charge between marmoset and human Trop-2.

*TROP2* ORF were synthesized for cynomolgus monkey, baboon, marmoset. These were transfected in recipient human HEK-293 and primate COS-7 cells. Flow cytometry analysis of transfectants was then performed for recognition of NHP Trop-2 by directly fluorochrome-conjugated Hu2G10 and Hu2EF. Trop-2 was efficiently recognized by Hu2G10 and Hu2EF in all tested NHP Trop-2 transfectants. Lower absolute intensity of binding was observed for marmoset Trop-2, in apparent consistency with a higher polymorphism of solvent-exposed residues. However, no human-revertant residue mutagenesis studies were performed to formally confirm these findings.

Taken together these findings supported the conservation of structure and function of Trop-2. We explored Trop-2 expression patterns in rhesus monkeys and found high levels of expression in multi-stratified epithelia of esophagus, tongue, skin and exocervix, together with multi-stratified urothelium. Additional expression was detected in different districts of the intestine, in kidney, lung, pancreas and most exocrine glands. Trop-2 conservation makes NHPs valuable models where to study Trop-2 function during development and in adult tissues, as well as reliable models for toxicity studies of Trop-2-targeting immunotherapy. Notably no cleaved forms of Trop-2 were detected, just as in human samples, indicating *in vivo* reliable assessment of discrimination between cancer-activated Trop-2 and uncleaved, low-to-nil binding Trop-2 structure. The Hu2G10 showed a high affinity of <1.0 × 10^−12^ M for cancer-specific, cleaved/activated Trop-2 but a ≈10,000-fold lower binding capacity for the normal-tissue expressed form.

A PK study was carried out in cynomolgus monkeys over 28 days following mAb infusion. Specific anti-idiotypic antibodies were generated to directly measure mAb concentrations in serum by tailored ELISA assays. Serum concentrations of Hu2G10 and Hu2EF peaked at 2 h post-intravenous injection and reached baseline levels by day 21, with a t_1/2_ of 6.5 and 5.5 days, respectively, at the maximum dose of 10 mg/kg. Thus, Hu2G10 and Hu2EF are stable in plasma and are detectable in the circulation up to 3 weeks after the infusion.

None of the tested cynomolgus monkeys showed significant neurological, respiratory, digestive and urinary symptoms. There was no body-weight loss in any of the monkeys during the 28-day study. No alterations of blood monocyte, neutrophil and basophil counts nor significant changes in biochemical parameters were detected.

Trop-2-specific antitumor activity of Hu2G10 in preclinical models was shown in prostate cancer (DU-145), colon cancer (HT29, KM12SM, HCT-116 U5.5), ovarian cancer (SKOV-3), pancreatic cancer (BxPc3), breast cancer (SKBr3), but not in Trop-2-nil tumor models (Guerra et al., 2023a). The 2EF mAb was demonstrated to bind Trop-2 at cell-cell junctions in MCF-7 breast cancer cells, and in deeply-seated sites in DU-145 prostate tumors, that were inaccessible to benchmark anti-Trop-2 antibodies. The 2EF antibody showed anticancer activity against SKOv3 ovarian, Colo205, HT29, HCT116 colon and DU-145 prostate tumors. Supported by different recognition modes of Trop-2 by 2EF and 2G10, synergy between 2EF and 2G10 against tumor xenotransplants was demonstrated, opening novel avenues for Trop-2-targeted therapy (Guerra et al., 2023b).

In previous studies, administration of lysosomally-targeted (DeVay et al., 2017) anti-Trop-2 mAb in NHP toxicity studies resulted in target-mediated effects in skin and oral mucosa, consistent with Trop-2 on-target/off-tumor toxicity in these epithelial tissues. Subsequently, the PF-06380101 was tested in a phase 1 study in patients with advanced solid tumors (King et al., 2018). Patients experienced neutropenia, skin rash and mucosal inflammation as dose limiting toxicities, indicating the broad Trop-2 expression in normal epithelia (Stepan et al., 2011; Trerotola et al., 2013a; Trerotola et al., 2021) as a key hurdle in Trop-2-targeted therapy, and showing distinct primate toxicity as a pivotal indicator for human studies.

Our findings rigorously demonstrate that multiple primate species are reliable models where to test PK and toxicity of novel Trop-2-targeting immunotherapies. In tested NHPs, Hu2G10 and Hu2EF showed favorable PK profiles and essential absence of toxicity. Thus Hu2G10 and Hu2EF mAbs are candidate to become valuable therapeutic tools in clinical settings with potentially high therapeutic index and efficacy over a broad range of Trop-2-expressing tumors.

## Data Availability

The original contributions presented in the study are included in the article/Supplementary Material, further inquiries can be directed to the corresponding author.

## References

[B1] Alberti S. Bucci C. Fornaro M. Robotti A. Stella M. (1991). Immunofluorescence analysis in flow cytometry: better selection of antibody-labeled cells after fluorescence overcompensation in the red channel. J. Histochem Cytochem 39 (5), 701–706. 10.1177/39.5.1901878 1901878

[B2] Alberti S. Herzenberg L. A. (1988). DNA methylation prevents transfection of genes for specific surface antigens. Proc. Natl. Acad. Sci. U. S. A. 85, 8391–8394. 10.1073/pnas.85.22.8391 3054885 PMC282463

[B3] Alberti S. Nutini M. Herzenberg L. A. (1994). DNA methylation prevents the amplification of TROP1, a tumor associated cell surface antigen gene. Proc. Natl. Acad. Sci. U. S. A. 91, 5833–5837. 10.1073/pnas.91.13.5833 8016075 PMC44091

[B4] Alberti S. Parks D. R. Herzenberg L. A. (1987). A single laser method for subtraction of cell autofluorescence in flow cytometry. Cytometry 8, 114–119. 10.1002/cyto.990080203 3556100

[B5] Ambrogi F. Fornili M. Boracchi P. Trerotola M. Relli V. Simeone P. (2014). Trop-2 is a determinant of breast cancer survival. PLoS One 9 (5), e96993. 10.1371/journal.pone.0096993 24824621 PMC4019539

[B6] Bardia A. Hurvitz S. A. Tolaney S. M. Loirat D. Punie K. Oliveira M. (2021). Sacituzumab govitecan in metastatic triple-negative breast cancer. N. Engl. J. Med. 384 (16), 1529–1541. 10.1056/NEJMoa2028485 33882206

[B7] Bardia A. Mayer I. A. Vahdat L. T. Tolaney S. M. Isakoff S. J. Diamond J. R. (2019). Sacituzumab govitecan-hziy in refractory metastatic triple-negative breast cancer. N. Engl. J. Med. 380 (8), 741–751. 10.1056/NEJMoa1814213 30786188

[B8] Bertoni M. Kiefer F. Biasini M. Bordoli L. Schwede T. (2017). Modeling protein quaternary structure of homo- and hetero-oligomers beyond binary interactions by homology. Sci. Rep. 7 (1), 10480. 10.1038/s41598-017-09654-8 28874689 PMC5585393

[B9] Calabrese G. Crescenzi C. Morizio E. Palka G. Guerra E. Alberti S. (2001). Assignment of TACSTD1 (alias TROP1, M4S1) to human chromosome 2p21 and refinement of mapping of TACSTD2 (alias TROP2, M1S1) to human chromosome 1p32 by *in situ* hybridization. Cytogenet Cell Genet. 92 (1-2), 164–165. 10.1159/000056891 11306819

[B10] Dell’Arciprete R. Stella M. Fornaro M. Ciccocioppo R. Capri M. G. Naglieri A. M. (1996). High-efficiency expression gene cloning by flow cytometry. J. Histochem Cytochem 44, 629–640. 10.1177/44.6.8666748 8666748

[B11] DeVay R. M. Delaria K. Zhu G. Holz C. Foletti D. Sutton J. (2017). Improved lysosomal trafficking can modulate the potency of antibody drug conjugates. Bioconjugate Chem. 28 (4), 1102–1114. 10.1021/acs.bioconjchem.7b00013 28151644

[B12] El Sewedy T. Fornaro M. Alberti S. (1998). Cloning of the murine *Trop2* gene: conservation of a PIP2-binding sequence in the cytoplasmic domain of Trop-2. Int. J. Cancer 75 (2), 324–330. 10.1002/(sici)1097-0215(19980119)75:2<324::aid-ijc24>3.0.co;2-b 9462726

[B13] Fornaro M. Dell'Arciprete R. Stella M. Bucci C. Nutini M. Capri M. G. (1995). Cloning of the gene encoding TROP-2, a cell-surface glycoprotein expressed by human carcinomas. Int. J. Cancer 62, 610–618. 10.1002/ijc.2910620520 7665234

[B14] Guerra E. Relli V. Ceci M. Tripaldi R. Simeone P. Aloisi A. L. (2022). Trop-2, Na+/K+ ATPase, CD9, PKCα, cofilin assemble a membrane signaling super-complex that drives colorectal cancer growth and invasion. Oncogene 41 (12), 1795–1808. 10.1038/s41388-022-02220-1 35132180

[B15] Guerra E. Trerotola M. Relli V. Lattanzio R. Ceci M. Boujnah K. (2023b). The 2EF antibody targets a unique N-terminal epitope of trop-2 and enhances the *in vivo* activity of the cancer-selective 2G10 antibody. Cancers 15 (14), 3721. 10.3390/cancers15143721 37509383 PMC10378344

[B16] Guerra E. Trerotola M. Relli V. Lattanzio R. Tripaldi R. Ceci M. (2023a). 3D-informed targeting of the Trop-2 signal-activation site drives selective cancer vulnerability. Mol. Cancer Ther. 22 (6), 790–804. 10.1158/1535-7163.MCT-22-0352 36921314

[B17] Guerra E. Trerotola M. Relli V. Lattanzio R. Tripaldi R. Vacca G. (2021). Trop-2 induces ADAM10-mediated cleavage of E-cadherin and drives EMT-less metastasis in colon cancer. Neoplasia 23 (9), 898–911. 10.1016/j.neo.2021.07.002 34320447 PMC8334386

[B18] Hsu E.-C. Rice M. A. Bermudez A. Marques F. J. G. Aslan M. Liu S. (2020). Trop2 is a driver of metastatic prostate cancer with neuroendocrine phenotype via PARP1. Proc. Natl. Acad. Sci. 117 (4), 2032–2042. 10.1073/pnas.1905384117 31932422 PMC6994991

[B19] Jumper J. Evans R. Pritzel A. Green T. Figurnov M. Ronneberger O. (2021). Highly accurate protein structure prediction with AlphaFold. Nature 596 (7873), 583–589. 10.1038/s41586-021-03819-2 34265844 PMC8371605

[B20] Kamble P. R. Patkar S. R. Breed A. A. Pathak B. R. (2021). N-glycosylation status of Trop2 impacts its surface density, interaction with claudin-7 and exosomal release. Arch. Biochem. Biophys. 714, 109084. 10.1016/j.abb.2021.109084 34774484

[B21] King G. T. Eaton K. D. Beagle B. R. Zopf C. J. Wong G. Y. Krupka H. I. (2018). A phase 1, dose-escalation study of PF-06664178, an anti-Trop-2/Aur0101 antibody-drug conjugate in patients with advanced or metastatic solid tumors. Invest. New Drugs 36 (5), 836–847. 10.1007/s10637-018-0560-6 29333575 PMC7519583

[B22] Mori Y. Akita K. Ojima K. Iwamoto S. Yamashita T. Morii E. (2019). Trophoblast cell surface antigen 2 (Trop-2) phosphorylation by protein kinase C α/δ (PKCα/δ) enhances cell motility. J. Biol. Chem. 294 (30), 11513–11524. 10.1074/jbc.RA119.008084 31177095 PMC6663864

[B23] Ocean A. J. Starodub A. N. Bardia A. Vahdat L. T. Isakoff S. J. Guarino M. (2017). Sacituzumab govitecan (IMMU-132), an anti-Trop-2-SN-38 antibody-drug conjugate for the treatment of diverse epithelial cancers: safety and pharmacokinetics. Cancer 123 (19), 3843–3854. 10.1002/cncr.30789 28558150

[B24] Okajima D. Yasuda S. Maejima T. Karibe T. Sakurai K. Aida T. (2021). Datopotamab deruxtecan, a novel TROP2-directed antibody–drug conjugate, demonstrates potent antitumor activity by efficient drug delivery to tumor cells. Mol. Cancer Ther. 20 (12), 2329–2340. 10.1158/1535-7163.MCT-21-0206 34413126 PMC9398094

[B25] Pagni M. Ioannidis V. Cerutti L. Zahn-Zabal M. Jongeneel C. V. Falquet L. (2004). MyHits: a new interactive resource for protein annotation and domain identification. Nucleic Acids Res. 32, W332–W335. 10.1093/nar/gkh479 15215405 PMC441617

[B26] Pavšič M. (2021). Trop2 forms a stable dimer with significant structural differences within the membrane-distal region as compared to EpCAM. Int. J. Mol. Sci. 22 (19), 10640. 10.3390/ijms221910640 34638982 PMC8508679

[B27] Querzoli P. Pedriali M. Rinaldi R. Lombardi A. R. Biganzoli E. Boracchi P. (2006). Axillary lymph node nanometastases are prognostic factors for disease-free survival and metastatic relapse in breast cancer patients. Clin. Cancer Res. 12 (22), 6696–6701. 10.1158/1078-0432.CCR-06-0569 17121888

[B28] Relli V. Trerotola M. Guerra E. Alberti S. (2018). Distinct lung cancer subtypes associate to distinct drivers of tumor progression. Oncotarget 9 (85), 35528–35540. 10.18632/oncotarget.26217 30473748 PMC6238974

[B29] Ripani E. Sacchetti A. Corda D. Alberti S. (1998). Human Trop-2 is a tumor-associated calcium signal transducer. Int. J. Cancer 76, 671–676. 10.1002/(sici)1097-0215(19980529)76:5<671::aid-ijc10>3.0.co;2-7 9610724

[B30] Stepan L. P. Trueblood E. S. Hale K. Babcook J. Borges L. Sutherland C. L. (2011). Expression of Trop2 cell surface glycoprotein in normal and tumor tissues: potential implications as a cancer therapeutic target. J. Histochem Cytochem 59 (7), 701–710. 10.1369/0022155411410430 21551320 PMC3201164

[B31] Stoyanova T. Goldstein A. S. Cai H. Drake J. M. Huang J. Witte O. N. (2012). Regulated proteolysis of Trop2 drives epithelial hyperplasia and stem cell self-renewal via beta-catenin signaling. Genes Dev. 26 (20), 2271–2285. 10.1101/gad.196451.112 23070813 PMC3475800

[B32] Studer G. Tauriello G. Bienert S. Biasini M. Johner N. Schwede T. (2021). ProMod3-A versatile homology modelling toolbox. PLoS Comput. Biol. 17 (1), e1008667. 10.1371/journal.pcbi.1008667 33507980 PMC7872268

[B33] Sun M. Zhang H. Jiang M. Chai Y. Qi J. Gao G. F. (2021). Structural insights into the cis and trans assembly of human trophoblast cell surface antigen 2. iScience 24 (10), 103190. 10.1016/j.isci.2021.103190 34693228 PMC8517388

[B34] Tagawa S. T. Balar A. V. Petrylak D. P. Kalebasty A. R. Loriot Y. Fléchon A. (2021). TROPHY-U-01: a phase II open-label study of sacituzumab govitecan in patients with metastatic urothelial carcinoma progressing after platinum-based chemotherapy and checkpoint inhibitors. J. Clin. Oncol. 39, 2474–2485. 10.1200/JCO.20.03489 33929895 PMC8315301

[B35] Trerotola M. Cantanelli P. Guerra E. Tripaldi R. Aloisi A. L. Bonasera V. (2013a). Up-regulation of Trop-2 quantitatively stimulates human cancer growth. Oncogene 32, 222–233. 10.1038/onc.2012.36 22349828

[B36] Trerotola M. Guerra E. Ali Z. Aloisi A. L. Ceci M. Simeone P. (2021). Trop-2 cleavage by ADAM10 is an activator switch for cancer growth and metastasis. Neoplasia 23 (4), 415–428. 10.1016/j.neo.2021.03.006 33839455 PMC8042651

[B37] Trerotola M. Jernigan D. Liu Q. Siddiqui J. Fatatis A. Languino L. (2013b). Trop-2 promotes prostate cancer metastasis by modulating β1 integrin functions. Cancer Res. 73 (10), 3155–3167. 10.1158/0008-5472.CAN-12-3266 23536555 PMC3655712

[B38] Tunyasuvunakool K. Adler J. Wu Z. Green T. Zielinski M. Žídek A. (2021). Highly accurate protein structure prediction for the human proteome. Nature 596 (7873), 590–596. 10.1038/s41586-021-03828-1 34293799 PMC8387240

[B39] Zanna P. Trerotola M. Vacca G. Bonasera V. Palombo B. Guerra E. (2007). Trop-1 are conserved growth stimulatory molecules that mark early stages of tumor progression. Cancer 110 (2), 452–464. 10.1002/cncr.22785 17559145

