# Supplementary material for: Phylogenetic conservation of Trop-2 across species—rodent and primate genomics model anti-Trop-2 therapy for pre-clinical benchmarks
Source: Front Genet. 2024 Jan 5;14:1297367. doi: 10.3389/fgene.2023.1297367 (PMC10797630; doi:10.3389/fgene.2023.1297367)
Supplement: Supplementary file 3 [file DataSheet1.PDF]

Supplementary Material

Supplementary Methods

Cells. The COS-7 cells, an SV-40 T antigen-transformed CV-1 African green monkey kidney cell line (Gluzman, 1981), were a kind gift by Leonard A. Herzenberg (Stanford University, CA). The NS-0 murine myeloma was obtained from the European Collection of Authenticated Cell Cultures (ECACC, Salisbury, Wiltshire, UK). The human 293T kidney cell line (DuBridge et al., 1987) was a kind gift by Bob DuBridge (Stanford University, CA). Frozen master stocks were prepared for each cell line within the first two months from receipt. Experiments were performed on cells that had been resuscitated from master stocks and passaged for no more than 6 months. Cell cultures were periodically checked for absence of mycoplasma contamination by PCR analyses and DAPI staining. The COS-7, 293T, NS-0 cell lines were maintained in DMEM medium supplemented with 10% fetal calf serum, 100 IU/ml penicillin and 100 µg/ml streptomycin (Euroclone, Milano, Italy). NS-0 cells were grown in serum-free Hybridoma SFM medium (Thermo Fisher Scientific, Waltham, MA) for antibody production.

Antibodies. The AbT16 (Fradet et al., 1984, Alberti et al., 1992), 2G10 (Alberti et al., 2022b, Alberti et al., 2022a), 2EF anti-Trop-2 monoclonal antibodies (mAb) were purified and fluorochrome-conjugated as described (Alberti et al., 1994). The humanized Hu2G10 and Hu2EF antibodies were generated as described (Guerra et al., 2023a, Guerra et al., 2023b).

Endotoxin measurement. Contaminating endotoxin in antibody solutions and reagents was measured with the quantitative LAL Chromogenic Endotoxin Quantitation Kit (Pierce-Thermo Scientific), using *Escherichia coli* endotoxin as standard. The reagents used for the *in-vivo* experiments were virtually endotoxin-free (endotoxin < 0.05 EU/ml).

Generation, expression, and characterization of hybridomas producing anti-idiotypic anti-Hu2G10 antibodies. A male C57BL/6 mouse was immunized according to the schedule:

Day 0 – 25 μg foot pad injection of Hu2G10-6

Day 7 – 25 μg foot pad injection of Hu2G10-6

Day 14 – 15 μg foot pad injection of murine 2G10

Day 21 – 15 μg foot pad injection of murine 2G10

Day 24 – Lymph nodes were harvested from both hind legs

Mouse lymphocytes isolated from the lymph nodes were fused with NS-0 cells, according to standard hybridoma fusion procedures. The following day DME media containing 10% fetal calf serum (HyClone, Logan, UT) and 1x HAT supplement (Sigma, St. Louis, MO) (HAT selection media) was applied. Approximately 6 days after the initiation of selection, supernatants were screened for binding to Hu2G10-6 in the presence of irrelevant humanized IgG1/kappa antibody, to eliminate the clones that bound nonspecifically to human IgG1/kappa.

ELISA plates were coated overnight at 4°C with 100 μl/well of 2 μg/ml Hu2G10-6 in PBS, washed with washing buffer (PBS containing 0.05% Tween 20), and blocked with 300 μl/well of blocking buffer (PBS containing 2% Skim Milk and 0.05% Tween 20) for 30 min at room temperature (RT). After one wash with washing buffer, 10 μl/well of test samples diluted in 90 μl/well ELISA buffer (PBS containing 1% Skim Milk and 0.025% Tween 20) and spiked with 8 μg/ml of irrelevant humanized IgG1/kappa antibody were applied to the ELISA plate. After incubation for 1 hr at RT and one wash with washing buffer, bound antibodies were detected using 100 μl/well of 1/2,000-diluted HRP-conjugated goat anti-mouse IgG polyclonal antibody (pAb) (SouthernBiotech, Birmingham, AL) in ELISA buffer. After incubation for 30 min at RT followed by one wash with washing buffer, color development was performed by adding 100 μl/well of ABTS substrate (AMRESCO, Solon, OH) and stopped with 100 μl/well of 2% oxalic acid. Absorbance was read at 405 nm.

Hybridoma clones producing antibodies that could bind to Hu2G10 were further expanded to 24-well plates. Specific binding to Hu2G10-6 was assessed by screening against a panel of various humanized IgG antibodies, in addition to Hu2G10-6 and chimeric 2G10 (Ch2G10), as described. 1D4-1 was the only clone to show positive binding to both Hu2G10-6 and Ch2G10, but not to the irrelevant humanized antibodies.

1D4-1 was expanded in Hybridoma SFM medium to a density of about $3x10^6$/ml, fed with 1/10 volume of 35 g/L of Cell Boost 4 (HyClone), and grown further until cell viability became less than 50%. Anti-Hu2G10 1D4-1 was purified from culture supernatants using protein A columns. The yield of anti-Hu2G10 1D4-1 was 5.5 mg from 0.5 L culture supernatant.

Binding of purified anti-Hu2G10 1D4-1 to Hu2G10 was assessed by ELISA. ELISA plate wells were coated overnight at 4°C with 100 µl/well of 1 µg/ml anti-Hu2G10 1D4-1 in PBS. After blocking with SuperBlock (Thermo Fisher Scientific, Waltham, MA), 1 µg/ml Hu2G10-6 was 3-fold serially diluted in ELISA buffer and added to wells. Likewise, 10% human serum was 3-fold serially diluted in ELISA buffer and added to control wells. Antibody binding to anti-Hu2G10 1D4-1was detected as described.

Hu2G10 bound to anti-Hu2G10 1D4-1 in a dose-dependent manner (see **Figure S5**), with an EC_{50} value of 5.5 ng/ml. To confirm that the anti-Hu2G10 1D4-1 was viable for PK studies, its binding to Hu2G10 was examined by ELISA in the presence of 2% human serum, as described above, except that the Hu2G10-6 and HRP-conjugated goat anti-human kappa pAb were diluted in SuperBlock. When serial dilutions of Hu2G10-6 were done in SuperBlock spiked with 2% human serum, binding of Hu2G10 to anti-Hu2G10 1D4-1 showed a dose-dependent sigmoidal curve with a detection limit of 1 ng/ml.

Generation, expression, and characterization of hybridomas producing anti-idiotypic anti-Hu2EF antibodies. A female Sprague-Dawley rat was immunized according to the schedule:

Day 0 – 25 µg foot pad injection of Hu2EF-7

Day 7 – 25 µg foot pad injection of Hu2EF-7

Day 14 – 15 µg foot pad injection of murine 2EF

Day 21 – 15 µg foot pad injection of murine 2EF

Day 24 – Lymph nodes were harvested from both hind legs

Rat lymphocytes isolated from the lymph nodes were fused with NS-0 cells, according to standard hybridoma fusion procedures. The following day HAT selection medium was applied. Approximately 10 days after the initiation of selection, supernatants were screened for binding to Hu2EF-7 in the presence of irrelevant humanized IgG1/kappa antibody to eliminate the clones that bound nonspecifically to human IgG1/kappa.

ELISA screening was performed as for anti-Hu2G10 clones, except that the coating antibody was Hu2EF-7 and the secondary antibody was HRP-conjugated polyclonal goat anti-rat IgG (Southern Biotech).

The anti-Hu2EF #36 was one of several clones that scored positive for binding to Hu2EF-7, but not to irrelevant humanized IgG antibodies. Anti-Hu2EF 36 was also chosen on the basis of higher expression levels than other clones, and was expanded in Hybridoma SFM medium to a density of about $3x10^6$/ml, fed with 1/10 volume of 35 g/L of Cell Boost 4 (HyClone), and grown further until the cell viability became less than 50%. Anti-Hu2EF 36 was purified from culture supernatants using protein A columns. The yield of anti-Hu2EF 36 was 8 mg from 0.5 L culture supernatant.

Binding of anti-Hu2EF 36 to Hu2EF or human serum was assessed by ELISA. ELISA plate wells were coated overnight at 4°C with 100 µl/well of 1 µg/ml anti-Hu2EF 36 in PBS, washed with washing buffer, and blocked with 300 µl/well of blocking buffer for 30 min at RT. After blocking, 1 µg/ml Hu2EF-7 was 3-fold serially diluted in ELISA buffer and added to wells. A polyclonal HRP-conjugated goat anti-human kappa (Southern Biotech) secondary antibody was used for detection. Binding of anti-Hu2EF 36 to human serum at 10% or lower was negligible. Hu2EF-7 bound to anti-Hu2EF 36 in a dose-dependent manner with an EC_{50} value of 85 ng/ml and a detection limit of several ng/ml (see **Figure S5**).

Supplementary Results

IHC analysis of Trop-2 expression in rodent tissues

The stratified squamous epithelium lining rodent esophagus and tongue showed no Trop-2 expression in the basal cells layer while a strong membrane immunoreactivity were present in the upper layers. The mouse major sublingual salivary glands expressed Trop-2 in a heterogeneous manner. Mucous and myoepithelial cells were Trop-2 negative, while the salivary ducts expressed low to high levels of Trop-2 in the intra- and inter-lobular ducts, respectively. The rat major sublingual salivary glands were Trop-2 negative. The gastric mucosa as well as the small bowel and colon mucosa did not express Trop-2. Expression of Trop-2 was not detected in liver hepatocytes as well as in the epithelium of the bile ducts and the gallbladder. The endocrine organs, e.g. thyroid follicular epithelium and adrenal cortex, were Trop-2 negative. Absence of Trop-2 expression was observed in the bronchial tree, bronchioles and alveolar epithelium cells. No membrane expression of Trop-2 was present in the renal glomerulum, renal tubules and the collecting ducts. No Trop-2 expression was detected in the ovary. The columnar epithelium lining the oviducts as well as the epithelium of the endocervix and the squamous epithelium of the exocervix all showed strong Trop-2 immunoreactivity. In the male genital tract Trop-2 expression was not present in the seminiferous tubules and interstitium. In the epithelial cells of the epididymis Trop-2 was predominantly detected in the lateral membrane. The glandular epithelium of the prostate showed a weak Trop-2 expression in the mouse, a strong one in the rat. Striated muscle cells of the skeletal system and the myocardium were Trop-2 negative. The cellular constituents of the central nervous system, including specialized tissues like the choroids plexus, were not stained for Trop-2. The expression of Trop-2 was barely or not detectable in the spleen and in the thymus.

The highest levels of Trop-2 were detected as associated to the cell membrane. A weaker Trop-2 intra-cellular staining was detected. This was due to synthesis in the ER, transport through the Golgi and accumulation in sub-membrane vesicles (Ambrogi et al., 2014). Only the terminal duct lobular unit of mammary glands, the mouse kidney's distal convoluted tubules, the rat sublingual salivary gland ducts showed Trop-2 cytoplasm immunoreactivity with low/nil membrane staining, suggesting differential regulation of Trop-2 transport in different tissues (**Figures S3, S4, Table S2**).

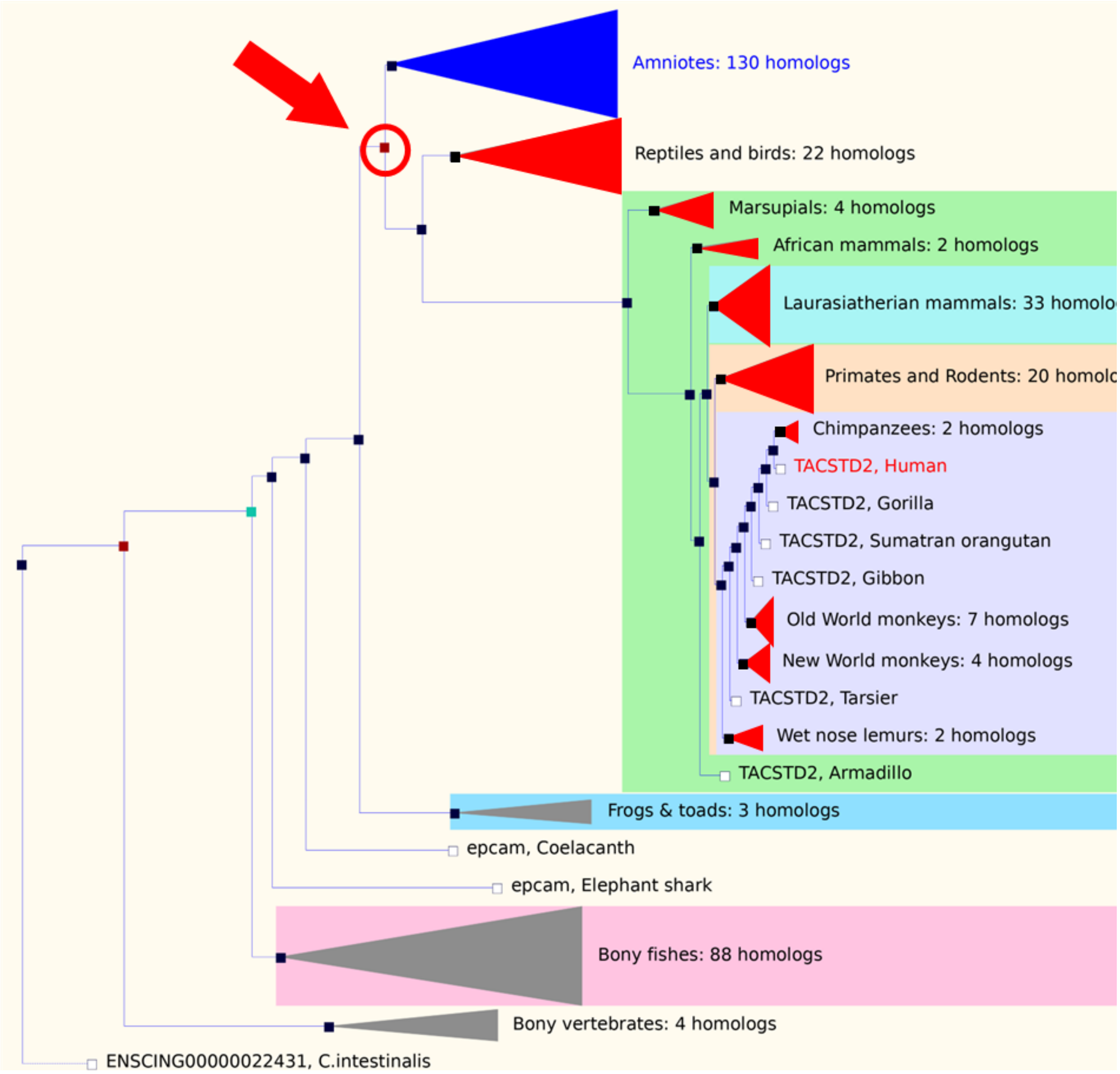

Supplementary Figure 1. Phylogenetic tree of the *TACSTD2/TROP2* gene. Gene Orthology/Paralogy prediction at https://www.ensembl.org was applied to trace human *TACSTD2/TROP2* evolution. Red squares at branching points represent duplications nodes, black squares represent speciation nodes, giving rise to paralogues and orthologues respectively. Ambiguous nodes are shown as green squares, gene nodes as white squares. Red triangles represent collapsed sub-nodes for *TACSTD2/TROP2*, the blue triangle represents the collapsed node for *TACSTD1/EPCAM*, gray triangles represents collapsed nodes for the single *TACSTD* gene. Red arrow: duplication node (circled in red) giving rise to *TACSTD1/EPCAM* and *TACSTD2/TROP2*.

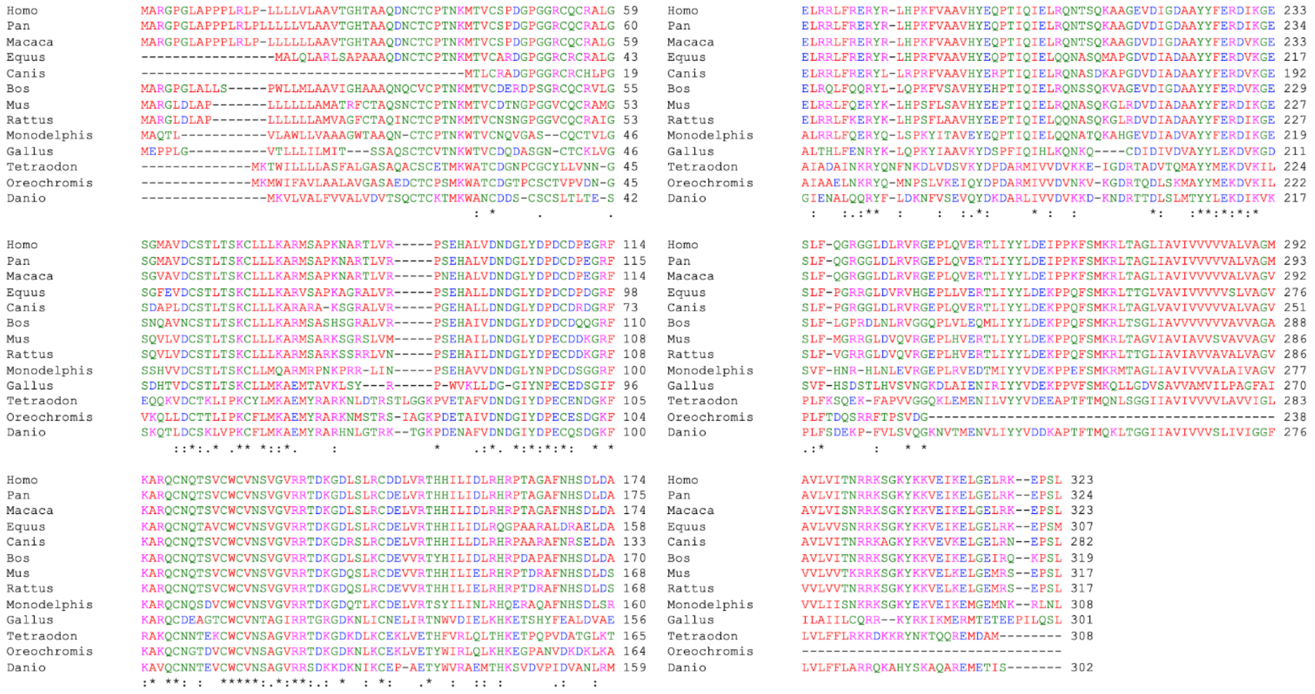

Supplementary Figure 2. Conservation of Trop-2 sequences in vertebrates. Vertebrate Trop-2 protein sequences were retrieved from http://www.ncbi.nlm.nih.gov/; http://blast.ncbi.nlm.nih.gov/. Protein sequence multialignments were performed with Clustal Omega (http://www.ebi.ac.uk/Tools/msa/clustalo/). Amino acid types are color-coded. Conservation levels are indicated below the sequences. Perfect conservation is indicated by <*>. Incomplete conservation but conservation of amino acid type is indicated by <:>. High but incomplete conservation is indicated by <.>.

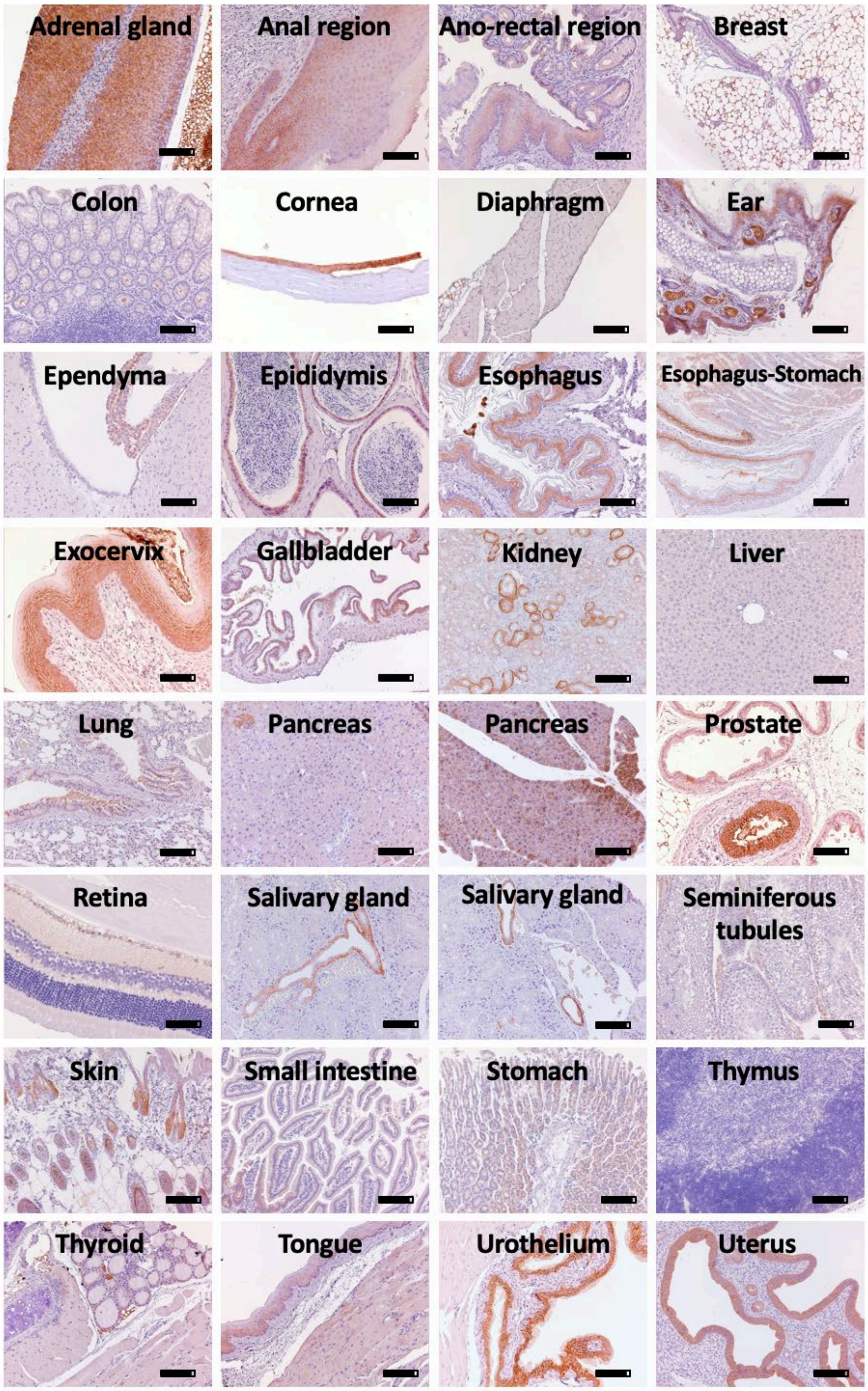

Supplementary Figure 3. IHC analysis of Trop-2 expression in the mouse. Staining was performed with the AF1122 anti-murine Trop-2 goat antiserum (R&D). Individual organs are indicated. Brown staining reveals Trop-2 expression. Bars: 50 µm.

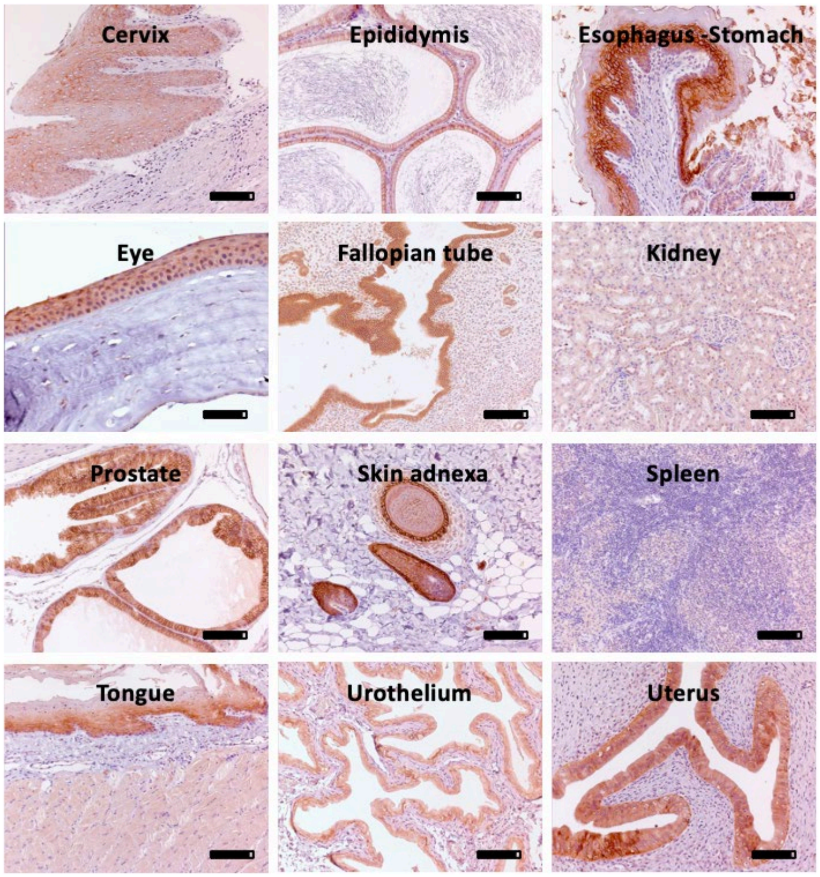

Supplementary Figure 4. IHC analysis of Trop-2 expression in the rat. Staining was performed with the AF1122 anti-murine Trop-2 goat antiserum (R&D). Individual organs are indicated. Brown staining reveals Trop-2 expression. Bars: 50 µm.

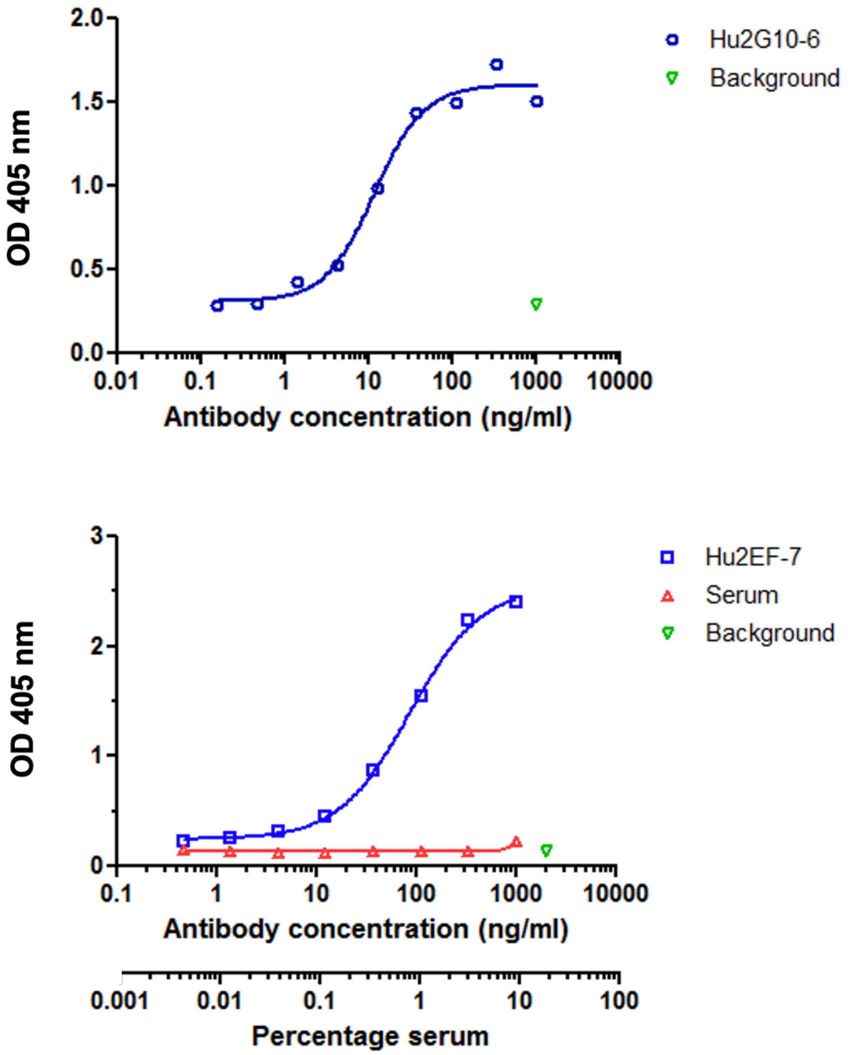

Supplementary Figure 5. (*top*) Binding of the anti-idiotypic 1D4-1 mAb to Hu2G10 in ELISA assay. Wells of an ELISA plate were coated overnight at 4°C with 100 µl/well of 1 µg/ml anti-Hu2G10 1D4-1 in PBS. After blocking with SuperBlock (Thermo Fisher Scientific), 1 µg/ml of Hu2G10-6 or 10% human serum in ELISA Buffer was applied, and serially diluted. Binding was detected using HRP-conjugated goat anti-human kappa pAb in ELISA buffer. Absorbance values (Y-axis) are plotted at each Hu2G10-6 concentration tested (X-axis, top line), and at each serum percentage (X-axis, bottom line) in the figure. Background: sample well in which all indicated procedures were performed, except no Hu2G10-6 was applied. (*bottom*) Binding of the anti-idiotypic Hu2EF #36 mAb to Hu2EF in ELISA assay. Wells of an ELISA plate were coated overnight at 4°C with 100 µl/well of 1 µg/ml anti-Hu2EF 36 in PBS. After blocking with Block Buffer, 1 µg/ml of Hu2EF-7 or 10% human serum in ELISA Buffer was applied, and serially diluted. Binding was detected using HRP-conjugated goat anti-human kappa pAb in ELISA buffer. Absorbance values (Y-axis) are plotted at each Hu2EF-7 concentration tested (X-axis, top line), and at each serum percentage tested (X-axis, bottom line) in the figure. Background: sample well in which all indicated procedures were performed, except neither Hu2EF-7 nor serum were applied.

Supplementary references

Alberti, S., Miotti, S., Stella, M., Klein, C. E., Fornaro, M., Ménard, S. & Colnaghi, M. I. 1992. Biochemical characterization of Trop-2, a cell surface molecule expressed by human carcinomas: formal proof that the monoclonal antibodies T16 and MOv-16 recognize Trop-2. *Hybridoma,* 11**,** 539-5.

Alberti, S., Nutini, M. & Herzenberg, L. A. 1994. DNA methylation prevents the amplification of TROP1, a tumor associated cell surface antigen gene. *Proc. Natl. Acad. Sci. USA,* 91**,** 5833-7.

Alberti, S., Trerotola, M. & Guerra, E. 2022a. Abstract 340: The Hu2G10 tumor-selective anti-Trop-2 monoclonal antibody targets the cleaved-activated Trop-2 and shows therapeutic efficacy against multiple human cancers. *Cancer Research,* 82**,** 340-340.

Alberti, S., Trerotola, M. & Guerra, E. 2022b. The Hu2G10 mAb targets the cleaved-activated form of Trop-2 and exploits vulnerability of multiple human cancers. *Journal of Clinical Oncology,* 40**,** e14548-e14548.

Ambrogi, F., Fornili, M., Boracchi, P., Trerotola, M., Relli, V., Simeone, P., La Sorda, R., Lattanzio, R., Querzoli, P., Pedriali, M., Piantelli, M., Biganzoli, E. & Alberti, S. 2014. Trop-2 is a determinant of breast cancer survival. *PLoS One,* 9**,** e96993.

Dubridge, R. B., Tang, P., Hsia, H. C., Leong, P. M., Miller, J. H. & Calos, M. P. 1987. Analysis of mutation in human cells by using an Epstein-Barr virus shuttle system. *Mol.Cell Biol.,* 7**,** 379-387.

Fradet, Y., Cordon-Cardo, C., Thomson, T., Daly, M. E., Whitmore Jr, W. F., Lloyd, K. O., Melamed, M. R. & Old, L. G. 1984. Cell-surface antigens of human bladder cancer defined by mouse monoclonal antibodies. *Proc. Natl. Acad. Sci. USA,* 81**,** 224-228.

Gluzman, Y. 1981. SV40-transformed simian cells support the replication of early SV40 mutants. *Cell,* 23**,** 175-182.

Guerra, E., Trerotola, M., Relli, V., Lattanzio, R., Ceci, M., Boujnah, K., Pantalone, L., Di Pietro, R., Iezzi, M., Tinari, N. & Alberti, S. 2023a. The 2EF Antibody Targets a Unique N-Terminal Epitope of Trop-2 and Enhances the In Vivo Activity of the Cancer-Selective 2G10 Antibody. *Cancers* [Online], 15.

Guerra, E., Trerotola, M., Relli, V., Lattanzio, R., Tripaldi, R., Ceci, M., Boujnah, K., Pantalone, L., Sacchetti, A., Havas, K. M., Simeone, P., Travali, N., Querzoli, P., Pedriali, M., Roversi, P., Iezzi, M., Tinari, N., Antolini, L. & Alberti, S. 2023b. 3D-informed targeting of the Trop-2 signal-activation site drives selective cancer vulnerability. *Mol Cancer Ther,* 22**,** 790-804.
